# Transcription factor binding site orientation and order are major drivers of gene regulatory activity

**DOI:** 10.1038/s41467-023-37960-5

**Published:** 2023-04-22

**Authors:** Ilias Georgakopoulos-Soares, Chengyu Deng, Vikram Agarwal, Candace S. Y. Chan, Jingjing Zhao, Fumitaka Inoue, Nadav Ahituv

**Affiliations:** 1 Department of Bioengineering and Therapeutic Sciences, University of California San Francisco, San Francisco, CA USA; 2 Institute for Human Genetics, University of California San Francisco, San Francisco, CA USA; 3 Institute for Personalized Medicine, Department of Biochemistry and Molecular Biology, The Pennsylvania State University College of Medicine, Hershey, PA USA; 4 mRNA Center of Excellence, Sanofi Pasteur Inc., Waltham, MA USA; 5 Institute for the Advanced Study of Human Biology (WPI-ASHBi), Kyoto University, Kyoto, Japan

**Keywords:** Gene regulatory networks, Gene regulation, Transcriptional regulatory elements

## Abstract

The gene regulatory code and grammar remain largely unknown, precluding our ability to link phenotype to genotype in regulatory sequences. Here, using a massively parallel reporter assay (MPRA) of 209,440 sequences, we examine all possible pair and triplet combinations, permutations and orientations of eighteen liver-associated transcription factor binding sites (TFBS). We find that TFBS orientation and order have a major effect on gene regulatory activity. Corroborating these results with genomic analyses, we find clear human promoter TFBS orientation biases and similar TFBS orientation and order transcriptional effects in an MPRA that tested 164,307 liver candidate regulatory elements. Additionally, by adding TFBS orientation to a model that predicts expression from sequence we improve performance by 7.7%. Collectively, our results show that TFBS orientation and order have a significant effect on gene regulatory activity and need to be considered when analyzing the functional effect of variants on the activity of these sequences.

## Abstract (web summary)

Gene regulatory grammar remains difficult to decipher, hindering our ability to link genotype to phenotype. Here they use massively parallel reporter assays to test over 200,000 synthetic sequences, finding that transcription factor binding site order and orientation have a major effect on gene regulatory activity.

## Introduction

Gene regulatory sequences are a major cause of human disease^1^. More than 90% of disease-associated variants are found in non-coding regions of the human genome^2–4^. However, the regulatory code remains only partially understood, precluding our ability to link variants in regulatory sequences with functional outcomes, such as disease. In addition, a better understanding of the regulatory code could allow us to synthetically design sequences that could drive therapeutic molecules in a beneficial spatio-temporal manner and provide favorable therapeutic expression levels for them^5^.

Combinatorial transcription factor binding is instrumental in organizing gene expression patterns across developmental time points, tissues and in disease^6^. For enhancers, two models of *cis*-regulatory syntax have been proposed and there is evidence supporting both. The first is the “enhanceosome model” which proposes that the precise orientation, positioning and order of TFBSs is required for an enhancer to function^7^. This model is inspired by the interferon-beta (IFN-beta) enhanceosome, which is highly conserved and for which an atomic model of cooperative transcription factor binding has been produced. The billboard model, also known as the information display model, suggests a more flexible architecture for enhancer grammar, in which the combination, orientation, order and distance of cognate motifs is not fixed, but instead can vary without impacting enhancer function and only the presence of the binding sites themselves is critical^8^. Utilizing consecutive affinity-purification systematic evolution of ligands by exponential enrichment (CAP-SELEX) experiments, 9,400 transcription factor-transcription factor–DNA interactions were functionally analyzed showing that both the orientation and distance between TFBSs determined heterodimer formation for a plethora of transcription factor pairs^9^. In addition, in previous work, we identified widespread transcriptional strand asymmetries in the orientation of TFBSs across human transcribed regions^10^, a research area in which there have been a few studies to date^11–15^.

Massively parallel reporter assays (MPRAs) provide the ability to measure thousands of sequences for their regulatory activity by placing these sequences in front of a transcribed barcode^16–18^. Utilizing MPRA, we previously designed a library of 5,000 sequences in which we tested the effect of TFBS copy number, spacing and order^19^. This study found that homotypic (combinations of same TFBS) and heterotypic (combinations of different TFBS) synthetic enhancers support a flexible model of regulatory element activity. Here, we utilized an MPRA library of over 200,000 sequences to comprehensively test the effects of strand orientation and order of homotypic and heterotypic TFBS clusters. We identified orientation and permutation preferences for the majority of the TFBS examined. We use these results to establish a set of *cis*-regulatory rules for the orientation of homotypic and heterotypic TFBSs. To corroborate our findings, we analyzed all human promoters and a human HepG2 MPRA dataset and identified similar TFBS strand asymmetries and order effects that influence expression levels. We next develop a predictive model of sequence activity which performs significantly better when TFBS orientation is taken into consideration. Overall, our study showcases the importance of strand orientation and order on regulatory activity and provides usage cases of how to utilize these features to increase our understanding of regulatory grammar and predict the effects of variants within these elements.

## Results

### MPRA library generation

To systematically test the effects of orientation and order of homotypic and heterotypic TFBSs, we designed an MPRA library utilizing different combinations of eighteen TFBSs. These include eleven transcription factors that are key regulators in the liver: AHR/ARNT, CEBPA, FOXA1, HNF1A, HNF4A, NR2F2, ONECUT1, PPARA, RXRA, TFAP2C and XBP1 and seven general transcription factors: CTCF, YY1, SP1, AP1, CREB1, REST and GABPA (Supplementary Table 1). Motifs were obtained from a previous HepG2 MPRA^19^ or JASPAR^20^ and HOCOMOCO^21^ databases (see more info on motif selection in **Methods**, Supplementary Table 1).The selected kmers for seventeen out of the eighteen TFBS were non-palindromic and could therefore be oriented relative to the transcription direction. CREB1 was the only palindromic (TGACGTCA) TFBS and was used as a control. We placed these TFBS onto two different neutral background sequences that are known to be negative for liver enhancer activity selected from a previous MPRA^19^ which were expanded from 167 to 200 base pairs (bp). This provided us with a longer background sequence to model our TFBS combinations. To validate that these extended 200 bp background sequences (#1: chr9:83,712,583-83,712,783 #2: chr2:211,153,222-211,153,422; hg19) do not have liver enhancer activity, we tested their ability to drive luciferase in HepG2 cells in the same vector used for lentivirus-based MPRA (lentiMPRA) (Supplementary Table 2). Both 200 bp sequences did not show enhancer activity compared to the empty vector (negative control), with background sequence 1 showing slightly higher activity compared to negative control and background sequence 2 being lower than the negative control (Supplementary Fig. 1a).

**Fig. 1:**
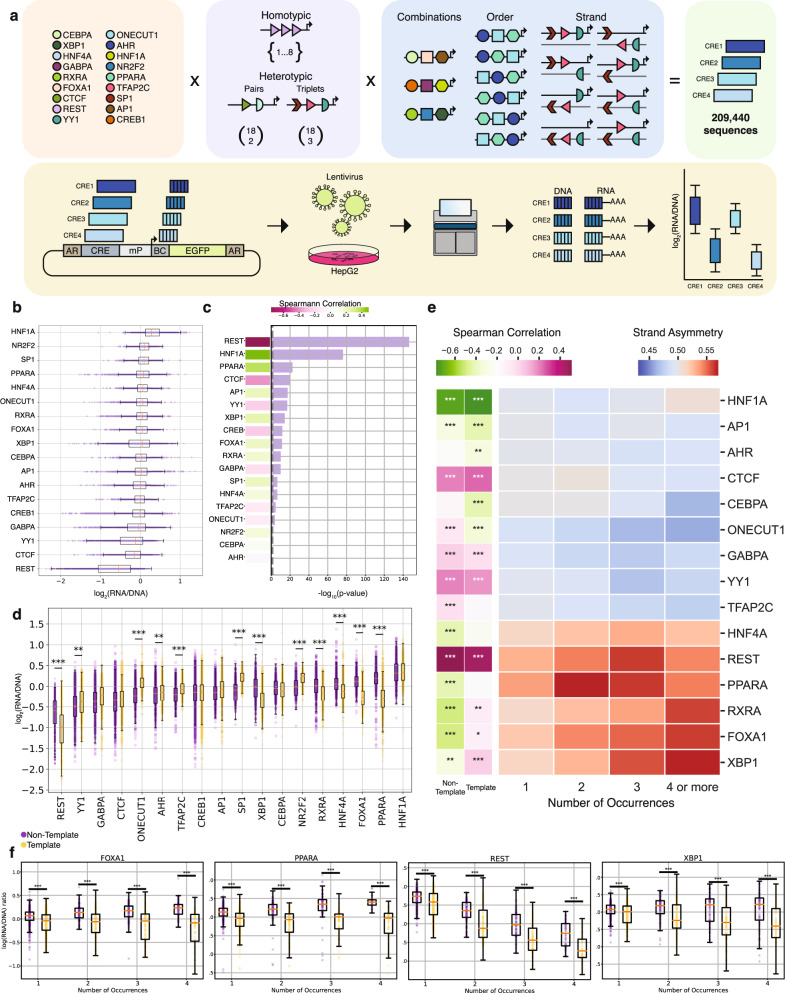
Homotypic TFBS orientation effects on *cis*-regulatory activity. **a**, Schematic of MPRA library design and workflow. Eighteen TFBSs, homotypic and heterotypic singlets, pairs and triplets were tested in all possible orientations and permutations. Homotypic TFBS insertions in the construct included 1-8 copies in either orientation and for heterotypic pairs and triplets permutations were tested in every TFBS orientation/combination. The lentiMPRA construct has a *cis*-regulatory element (CRE), minimal promoter (mP), barcode (BC), Enhanced Green Fluorescent Protein (EGFP) reporter and antirepressors (AR). Lentivirus was generated, cells infected and DNA and RNA barcodes sequenced. **b**, Expression levels of sequences harboring each TFBSs, for *n* = 2 background sequences. **c**, Barplot showing the -log(p-values) of the Spearman correlation between the number of occurrences and the mean expression values. Dotted gray line represents the threshold for Bonferroni corrected p-values of 0.05 (two-sided). Spearman correlation score is shown on the left. **d**, Expression levels for sequences with one or more TFBS occurrences at template or non-template orientation in yellow and purple, respectively, for *n* = 2 background sequences. Tiles contain non-template copies of TFBS or only template copies. Strand asymmetry was calculated as the ratio of the mean expression for sequences with TFBSs over both orientations (two-sided t-test and Bonferroni-corrected p-values). **e**, Heatmap showing the ratio of mean expression at the non-template over the template strand as a function of TFBS copy number. Spearman correlation between the number of occurrences and mean expression levels for TFBSs in the template and non-template shown as two-column heatmap (Spearman correlation with Bonferroni-corrected p-values (two-sided)). **f**, Association between expression levels and number of TFBS occurrences at the template and non-template orientation for REST, PPARA, FOXA1 and XBP1, for *n* = 2 background sequences (two-sided t-test and Bonferroni-corrected p-values). Adjusted p-values displayed as * for p-value<0.05, ** for p-value<0.01 and *** for p-value<0.001. In the boxplots, the median is indicated as the center line, the lower and upper limits are first quantile (25^th^ percentile) and third quantile (75^th^ percentile) respectively, the lower and upper whiskers are the lowest and maximum value of the data within 1.5 times the interquartile range over the 25^th^ and the 75^th^ percentile.

The designed library consisted of: 1) one, two, three, four, five, six and eight occurrences of homotypic TFBSs; 2) all 306 heterotypic pair permutations of the eighteen selected TFBSs; 3) all 4,896 heterotypic triplet permutations of the eighteen TFBSs (Fig. 1a). Every selected set of TFBSs was tested in all possible orientation combinations, which for single TFBSs were the template and non-template orientations, for pairs they were the four possible orientation combinations and for triplets they were the eight possible orientation combinations. For all single and pair combinations, three different distances of 5 bp, 10 bp or the most frequent genomic distance in the human genome between TFBSs were used. The latter was calculated by identifying the genomic locations of every TFBS and measuring the frequency of each pairwise distance (**Methods**). For triplet combinations, only the most frequent genomic distance between TFBSs across the human genome was tested, totaling 92,208 sequences (Supplementary Fig. 1b). This resulted in a library of 209,440 sequences.

The library was oligosynthesized and cloned into a lentiMPRA vector^22^. Virus was generated and HepG2 cells were infected, all following techniques described in^23^ (Fig. 1a). Following three days, to allow for viral integration and elimination of the majority of non-integrating virus, cells were harvested, DNA and RNA was generated, and barcodes sequenced. MPRA was conducted with three independent replicates and analyzed using MPRAflow^23^ which found all replicates to be correlated (Pearson correlation (r) between replicates 0.74-0.79; Supplementary Fig. 1c). Analysis of results between the two background sequences also showed correlation (Spearman correlation, rho=0.411, p-value<0.0001; Supplementary Fig. 1d), with sequences in background sequence 2 having lower activity and lower signal to noise ratio, likely due to the lower baseline activity of this background sequence. For subsequent analyses, we combined the results from both background sequences unless indicated otherwise.

### TFBS copy number correlates with expression

We first analyzed whether the number of homotypic TFBSs influences expression levels. The highest expression levels for a single TFBS was observed with the insertion of HNF1A, NR2F2 or SP1 and the lowest expression was observed with a single copy of either REST, YY1 or CTCF (Fig. 1b). HNF1A was shown to provide robust activation in HepG2 in our former MPRA^19^ and REST has been previously shown to act as a repressor element^24^, consistent with our results.

We next investigated if increasing the number of copies of homotypic TFBSs influenced expression levels. For fourteen transcription factors (AP1, CEBPA, CREB1, CTCF, FOXA1, GABPA, HNF1A, HNF4A, PPARA, REST, SP1, TFAP2C, YY1, XBP1), we observed an association between the number of TFBS copies and expression levels. Among them, for six transcription factors (CREB1, CTCF, GABPA, REST, TFAP2C and ΥΥ1), we observed a negative correlation between having more copies and expression levels, whereas for the others we observed a positive correlation (Fig. 1c; Spearman correlation p-value<0.05, Bonferroni-corrected p-values). REST showed the largest decrease in expression levels between placing a single and four copies or more copies (44.2% decrease), while HNF1A showed the largest increase in expression levels due to multiplicity of TFBSs (25.1% increase) (Supplementary Fig. 1e**)**. In summary, we observe that increasing the number of homotypic TFBSs has an effect on regulatory activity for most TFBS.

### TFBS orientation significantly impacts expression

TFBSs can be found in two possible orientations relative to gene direction: 1) the transcribed template or 2) the non-template. We examined if the orientation of individual TFBSs influences expression levels. Out of the seventeen non-palindromic TFBS sequence motifs examined, twelve (AHR, FOXA1, HNF4A, NR2F2, ONECUT1, PPARA, REST, RXRA, SP1, TFAP2C, XBP1, YY1) displayed significant differences in expression depending on their orientation (Fig. 1d, Supplementary Fig. 2; t-test, Bonferroni corrected). The strongest orientation-dependent expression difference was observed for PPARA which showed 21% higher activity in the template versus the non-template orientation (t-test, Bonferroni corrected, p-value<0.001 on both negative constructs). We observed an association between the GC-skew levels of the TFBS and the orientation-dependent expression difference (Kendall Tau test, τ = 0.4, p-value<0.05), while no association was observed for the AT-skew levels (Kendall Tau test>0.05). We also found the variance between the two orientations differs significantly for 13 of the examined TFBSs, namely AHR, CEBPA, CTCF, FOXA1, GABPA, HNF4A, ONECUT1, PPARA, REST, SP1, TFAP2C, YY1 and XBP1 (Levene test, p-value<0.05, Bonferroni corrected p-values).

We next examined if multiple homotypic TFBS occurrences, all of which were inserted either in the template or in the non-template orientation, expand the observed differences in expression between the two orientations. We observed an augmented expression difference for a number of TFBSs in the template or in the non-template orientations (Fig. 1e, Supplementary Fig. 3). For seventeen TFBSs (with the exception of the palindromic CREB1), having four TFBS copies in either orientation led to significantly higher expression differences between template and non-template than a single TFBS (Monte Carlo simulations, p-value<0.05). As an example, for PPARA we observed a 41% activity difference when having four copies on the template versus the non-template orientation compared to 21% when only having one (t-test, Bonferroni-corrected p-values, p-value<0.05).

We also observed that for some TFBSs having more copies only increased transcriptional activity if they were on a specific orientation. For two TFBSs (AHR and CEBPA), the number of copies on the template strand was correlated with higher expression but not on the non-template (Fig. 1f; Spearman correlation, Bonferroni corrected p-values, p-value<0.05). For another three TFBSs, the number of copies on the non-template strand was positively (PPARA, HNF4A) or negatively (TFAP2C) correlated with higher expression levels, but not on the template. For ONECUT1, we observed that having more copies on the non-template strand increased expression while on the template strand this reduced expression (Fig. 1e; Spearman correlation, Bonferroni corrected, p-value<0.05), while for FOXA1, RXRA and XBP1, we observed the opposite (multiple copies on template strand increased expression and reduced expression in the non-template strand) (Fig. 1e-f; Spearman correlation, Bonferroni corrected, p-value<0.05). Collectively, these results show that for certain TFBSs, the number of homotypic copies can have a strong effect on transcription levels and this effect could be significantly dependent on orientation.

### TFBS orientation and position impact regulatory activity

**Fig. 2:**
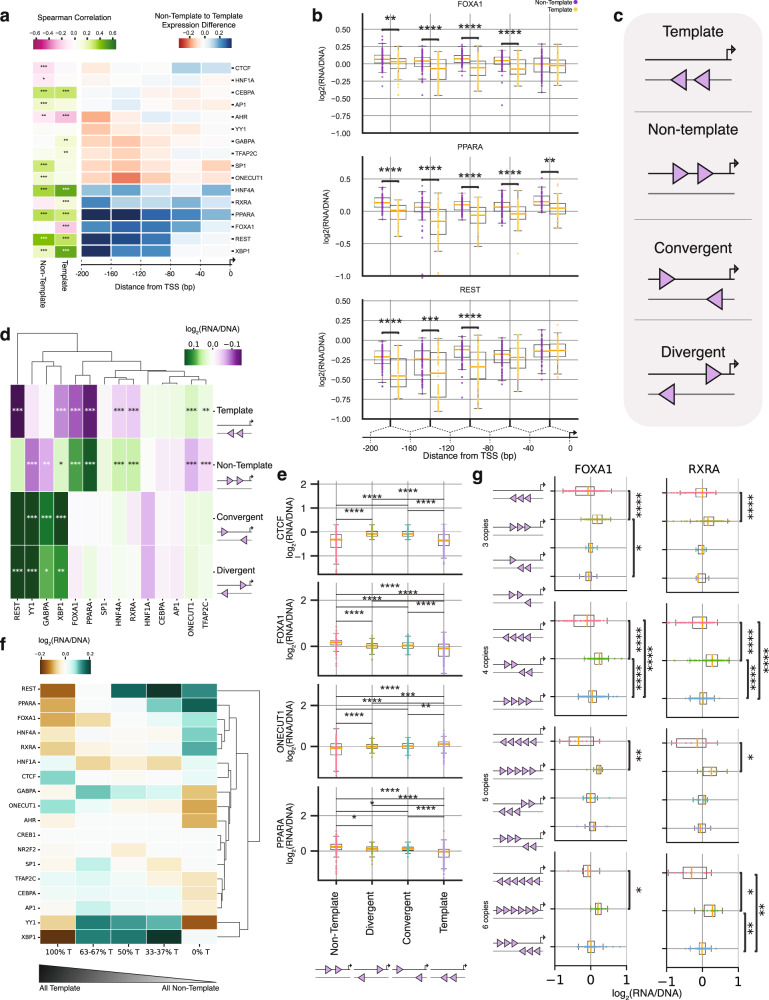
Permutation and orientation effects of heterotypic TFBS pairs. **a**, Heatmap showing the association between the distance in the MPRA tile (in bps) and expression levels for individual TFBSs. The Spearman correlation between distance and expression levels in TFBSs in the template, non-template and both template and non-template (combined) orientations is shown and the p-value was Bonferroni corrected. Non-template to template expression difference is shown as a function of distance from the transcription start site (TSS). **b**, Expression of PPARA, FOXA1 and REST TFBSs in the template and non-template orientation as a function of distance from TSS, for *n* = 2 background sequences. Statistical significance was calculated with a two-sided t-test and Bonferroni-corrected p-values. **c**, Schematic showing the four possible orientations for homotypic TFBS pairs: template, non-template, convergent and divergent. **d**, Heatmap showing the percent expression difference in each of the four possible orientations relative to the total expression levels. Statistical significance was estimated with two-sided t-test and Bonferroni-corrected p-values. **e**, Examples of CTCF, FOXA1, ONECUT1, PPARA, REST, YY1 and XBP1 displaying statistically significant bias in the expression between any two of the four possible orientations. Results obtained from *n* = 2 background sequences. **f**, The proportion of homotypic clusters of each TFBS in template and non-template orientation influences expression levels measured as fold-change from mean expression. Only TFBS for which sufficient barcodes were recovered are displayed. **g**, RXRA and FOXA1 expression difference between orientations for three, four, five and six copies. Results obtained from *n* = 2 background sequences. Adjusted p-values are displayed as * for p-value<0.05, ** for p-value<0.01 and *** for p-value<0.001. In the boxplots, the median is indicated as the center line, the lower and upper limits of the boxplots indicate the first quantile (25^th^ percentile) and the third quantile (75^th^ percentile) respectively, the lower and upper whiskers are the lowest and the maximum value of the data that are within 1.5 times the interquartile range over the 25^th^ and the 75^th^ percentile respectively.

We next analyzed whether the distance of TFBS from the TSS along with their orientation affects regulatory activity. We first analyzed this for a single TFBS copy. For nine TFBSs (AHR, CREB1, CTCF, GABPA, HNF1A, REST, SP1, XBP1 and YY1), we observed a positive correlation between the position of the TFBS and expression levels, with TFBS closer to the TSS providing higher transcriptional levels. Among these, CTCF and REST had the strongest correlation between position and expression, i.e., being closer to TSS led to stronger expression levels (33.6% increase for CTCF and 63.6% for REST in the −40bp to 0 bp bin, relative to the −200bp to −160bp bin), while two TFBS showed a negative correlation between position and expression (FOXA1 and ONECUT1 with 4.2% and 3.1% decrease in expression in the −40bp to 0 bp bin, relative to the −200bp to −160bp bin), (Supplementary Fig. 4; Spearman correlation, Bonferroni corrected p-values, p-value<0.05). We next investigated if the orientation (template or non-template) of the TFBS along with distance from the TSS affects transcriptional activity. For seven transcription factors (AP1, CEBPA, HNF4A, PPARA, REST, SP1 and XBP1), we observed a positive correlation between their distance from the TSS and significant expression difference between the two orientations, with TFBSs being farther away from the TSS showing a stronger effect (Fig. 2a-b, Supplementary Fig. 5). One such example was REST, where we observed that the expression difference between the two orientations decreased with proximity to the TSS and at the closest positions to the TSS there were no differences between the two orientations (Fig. 2b, Supplementary Fig. 5). We conclude that the orientation of a single TFBS can significantly influence transcription and the effect is associated with their position relative to the TSS.

### Orientation effects of multiple copies of homotypic TFBSs

We next investigated how the orientation of two homotypic TFBS copies affects transcriptional activity. Dimers for the same TFBS can be found in four possible orientations: 1) both at the template orientation; 2) both at the non-template orientation; 3) at divergent orientation; and 4) at convergent orientation (Fig. 2d). All four orientations were tested with the same inter-motif distances for pairs of TFBS. We found substantial biases in expression depending on dimer orientation for ten TFBS (FOXA1, GABPA, HNF4A, ONECUT1, PPARA, REST, RXRA, TFAP2C, XBP1 and YY1), with some differences leading to more than 20% changes in expression levels (Fig. 2e). For example, REST, XBP1 or YY1 in divergent and convergent orientations showed significantly higher expression than YY1 pairs or XBP1 pairs in template or non-template orientations (t-test, Bonferroni corrected p-values, p-value<0.001). For FOXA1, PPARA and RXRA two copies at the template orientation had the highest expression, whereas for ONECUT1 a dimer at the non-template provided the highest expression (Fig. 2e; t-test, Bonferroni-corrected p-values, p-value<0.001). Therefore, out of the four possible orientations in which homotypic TFBS pairs can be found at, for most TFBSs there is an optimal orientation between the two copies.

We next tested how additional TFBS copies of three, four, five or six motif occurrences of the same TFBS (homotypic) affect expression. For FOXA1, PPARA and RXRA the highest expression was observed with all copies being at the template orientation, whereas for CEBPA, ONECUT1 and TFAP2C the highest expression was reached with all copies being at the non-template orientation (Fig. 2f-g). For four TFBSs, GABPA, REST, XBP1 and YY1, sequences with equal or close to equal number of TFBSs in the two orientations had the highest expression levels, which is consistent with our aforementioned findings in which divergent and convergent orientations displayed the highest expression for these transcription factors (Fig. 2g). Together, these results suggest that TFBS orientation has a major effect on regulatory element activity.

### The order of heterotypic TFBS pairs impacts expression

We next analyzed our results for heterotypic TFBSs. We first assessed how combinations of heterotypic TFBS pairs and their order influence expression levels. We analyzed all possible TFBS pair combinations of the eighteen TFBSs and found clear preferences for specific pairs (Supplementary Fig. 7). Amongst all pairs, the interaction between HNF1A and AHR displayed the highest expression levels. The lowest expression levels were found for the combination of CTCF and REST, being even more repressive than a homodimer for each one separately (Supplementary Fig. 7).

We next investigated if these TFBS pairs displayed significant expression differences depending on their order. For each heterotypic TFBS pair, the TFBS that was closest relative to the TSS was termed “Closest” and the most distant was termed “Distant”. With the exception of HNF4A, the position of each TFBS as either”Closest” or “Distant” influenced expression for all TFBSs (t-test, Bonferroni corrected; Supplementary Fig. 8d), indicating that the order in which TFBSs are placed is important.

**Fig. 3:**
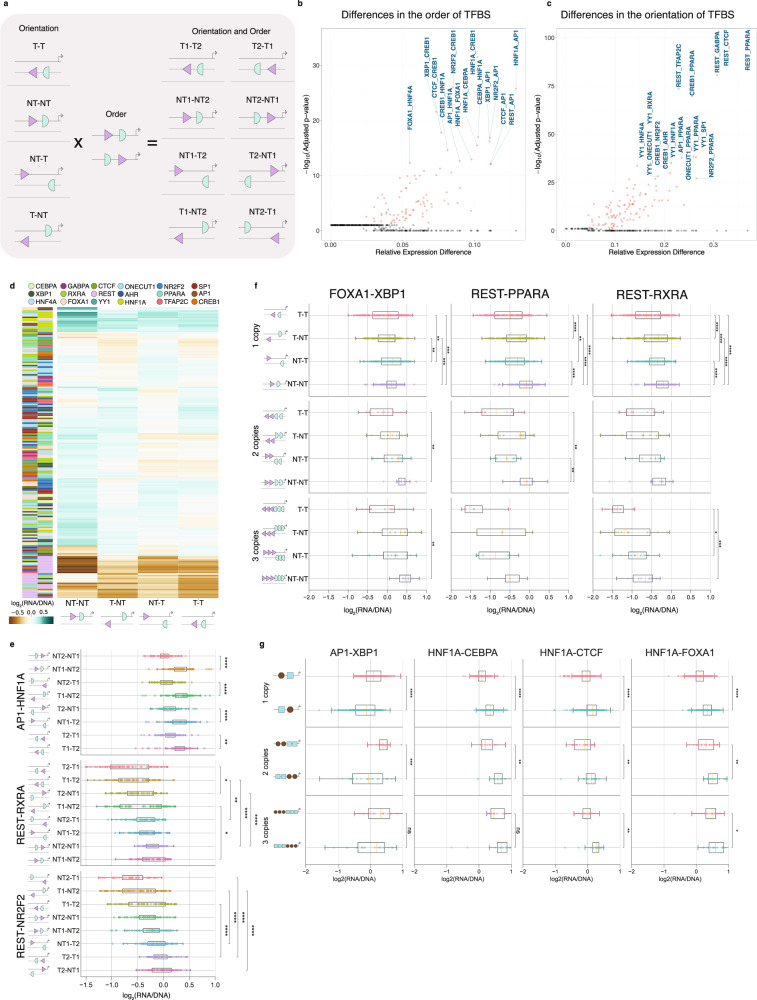
Heterotypic TFBSs show significant orientation dependence. **a**, Schematic showing the four possible orientations for two heterotypic TFBSs, both non-template (NT-NT), both template (T-T), convergent and divergent orientations. **b**, Mean expression difference between sequences with TFBS pairs with different orders with highest and lowest expression levels and associated adjusted p-value from two-sided t-tests with Bonferroni-corrected p-values. **c**, Mean expression difference between sequences with TFBS pair orientations with highest and lowest expression levels and associated adjusted p-value from two-sided t-tests with Bonferroni-corrected p-values. **d**, The orientation of TFBSs in heterotypic TFBS pairs influences expression levels. Only pairs with adjusted p-value<0.05 are shown (one-way ANOVA, Bonferroni corrected). Only sequences with a single copy of each TFBS in the heterotypic TFBS pair are shown. The combination of Template (T) and Non-template (NT) orientations for both TFBSs are shown in y-axis. **e**, Examples displaying statistical significant expression difference between the four possible orientations for REST-HNF4A, REST-RXRA, CREB1-RXRA and FOXA1-RXRA. Results obtained from *n* = 2 background sequences. Statistical significance was estimated with t-tests with Bonferroni-corrected p-values. **f**, Dosage-dependent effects of the proportion of template (T) and non-template (NT) TFBSs in heterotypic clusters for three heterotypic TFBS pairs, AHR-PPARA, CTCF-HNF4A and AP1-PPARA. Statistical significance was estimated with two-sided t-tests and Bonferroni correction. Results obtained from *n* = 2 background sequences. **g**. The order with which TFBS sites are placed for TFBS pairs significantly influences expression. Examples shown for AP1-XBP1, HNF1A-CEBPA, HNF1A-CTCF and HNF1A-FOXA1. Results obtained from *n* = 2 background sequences. Statistical significance was estimated with two-sided t-tests and Bonferroni correction. Adjusted p-values displayed as * for p-value<0.05, ** for p-value<0.01 and *** for p-value<0.001. In the boxplots, the median is indicated as the center line, the lower and upper limits of the boxplots indicate the first quantile (25^th^ percentile) and the third quantile (75^th^ percentile) respectively, the lower and upper whiskers are the lowest and the maximum value of the data that are within 1.5 times the interquartile range over the 25^th^ and the 75^th^ percentile respectively.

We then assessed whether the order of the heterotypic TFBS pairs relative to the TSS affects gene expression (Fig. 3a). There were 89 transcription factor pairs (representing ~29% of the interactions) that showed highly significant differences in expression depending on TFBS order (Fig. 3b-e; t-test, Bonferroni corrected). The main TFBSs which showed a strong effect on order when paired with different TFBSs were CREB1, HNF1A and XBP1. Combinations that had a major effect on expression with these TFBSs included XBP1-CTCF, XBP1-AP1, HNF1A-CREB1, NR2F2-CREB1, CEBPA-HNF1A and HNF1A-FOXA1 among others (Fig. 3b). We also note that for multiple TFBS pairs (58%), there were significant biases between the two possible orders (Levene test, p-value<0.05, Bonferroni corrected p-values). These results suggest that the order of heterotypic TFBS pairs can significantly influence regulatory activity.

### The orientation of heterotypic TFBS pairs impacts expression

We analyzed the orientation of individual TFBSs and examined how it influenced the expression levels of TFBS pairs. For several TFBSs, the orientation of their cognate TFBSs substantially influenced expression levels (Supplementary Fig. 8a-b). All seventeen non-palindromic TFBSs were found to have different expression levels in the template and non-template orientations across their TFBS pair interactions (Supplementary Fig. 8c; t-test with Bonferroni-corrected p-values, p-value<0.05), which was in line with what was observed previously for them individually (Fig. 1d, Supplementary Data Fig. 2).

Taking into account strand orientation irrespective of order, each of the two TFBSs of a heterotypic pair can have four possible configurations (two orientations for each TFBS) (Fig. 3a). We investigated if the orientation of the TFBSs impacted expression levels irrespective of TFBS order. Overall, we found 111 transcription factor pairs (representing ~36% of the total interactions; One-way ANOVA, Bonferroni-corrected) showing highly significant differences in expression due to orientation (Fig. 3c-d, Supplementary Data 1). We also note that 34 of these also showed statistically significant differences depending on the order (Fig. 3b). Among the top pairs displaying the biggest differences in expression due to changes in orientation were REST-PPARA, REST-TFAP2C, REST-CTCF and YY1-RXRA (Fig. 3c-d). Interestingly, from the set of TFBS pairs for which we recovered sufficient barcodes for all four orientation configurations, 72% of TFBS pairs showed significant differences in expression due to the orientation. This result suggests pervasive differences in expression due to changes in orientation for heterotypic TFBS pairs.

### Heterotypic TFBS order and orientation impact expression

**Fig. 4:**
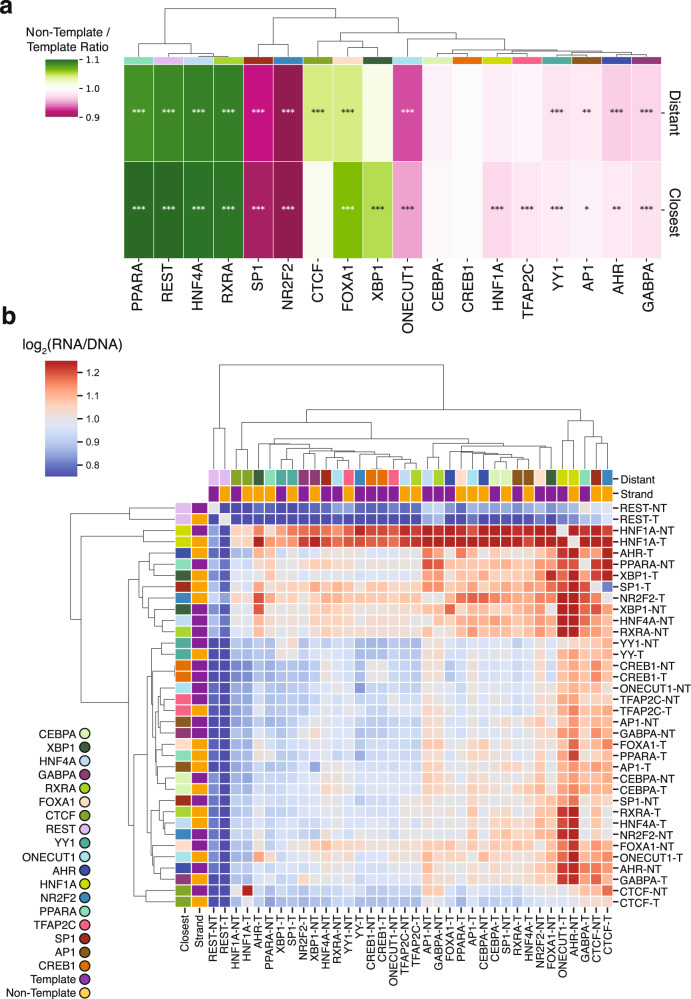
The orientation and order of heterotypic TFBSs impacts expression levels. **a**, Hierarchical clustering of TFBSs for each TFBS showing the mean non-template to template expression ratio. “Distant” refers to the TFBS being most distant relative to the TSS and “Closest” to the TFBS being closest to the TSS as part of the heterotypic TFBS pairs. Enrichment was estimated as the mean non-template to mean template expression. Statistical significance was estimated with two-sided t-test and Bonferroni corrections calculated between the template and non-template occurrences of each TFBS for “Closest” and “Distant” positions separately. **b**, Hierarchical clustering of TFBS pairs in the non-template (NT; purple) or template (T; yellow) orientation. The order of transcription factors is denoted with the most distant transcription factor relative to the TSS shown in the rows. In the first column, each color represents a different transcription factor. Adjusted p-values displayed as * for p-value<0.05, ** for p-value<0.01 and *** for p-value<0.001.

Next, we examined if the orientation and order in which heterotypic TFBSs are located influences regulatory activity. For sixteen out of eighteen TFBSs (the two that did not show this are CEBPA and CREB1), there were significant differences in expression depending on their orientation across the heterotypic TFBS pairs irrespective of the order (proximal or distant) (Fig. 4a; t-test with Bonferroni-corrected p-values). More specifically, for AHR, GABPA, HNF4A, NR2F2, ONECUT1, PPARA, REST, SP1 and YY1, there were significant expression differences depending on the orientation irrespective of the TFBS position in the heterotypic TFBS pair (proximal or distant) (t-test with Bonferroni-corrected p-values, p-value<0.05). For CTCF, HNF1A, TFAP2C and XBP1, order was an important variable in the observed orientation effects (Fig. 4a). The pairwise comparisons for all heterotypic TFBS pairs also indicated widespread differences depending on the orientation across individual TFBS pairs (Fig. 4b). This was particularly evident for FOXA1, NR2F2, ONECUT1, PPARA, SP1 and XBP1, whose TFBSs displayed marked expression differences depending on the combination of TFBS orientations in the template and non-template strand. These results indicate that the orientation and order of heterotypic TFBSs can have a significant effect on *cis*-regulatory activity.

Finally, we examined what proportion of heterotypic TFBS pairs displayed both orientation and order preferences. We found a total of 33 TFBS pairs (representing 10% of pairs) for which both orientation and order were significantly associated with expression. From the eight possible orientation and order combinations for heterotypic TFBS pairs, we found differences in expression up to 81% depending on specific combinations of order and orientation. Certain TFBSs displayed a stronger effect between different TFBS orders, but not for different orientations (Fig. 3e, Supplementary Fig. 9). For example, the TFBS pair of AP1-HNF1A showed only order-specific effects, having a 39% difference in expression depending on TFBS order, but the orientation did not have an effect on regulatory activity (Fig. 3e). Other TFBS pairs showed stronger differences in expression when changing the orientations but not the order. As an example, the TFBS pair of REST-RXRA showed primarily orientation-specific effects (i.e., a 64% difference), but non-significant effects of order changes (Fig. 3e). Certain pairs, such as REST-NR2F2, showed dependence on both orientation and order (i.e., an 80% difference, Fig. 3e). These results are consistent with our earlier homotypic TFBS observations, showing that orientation has a major effect on regulatory activity and that the order of some heterotypic TFBS can further impact this effect.

### Heterotypic TFBS optimal grammar is independent of the number of copies

We examined how the orientation of multiple copies of heterotypic TFBS pairs affect their regulatory activity, by having one, two or three consecutive copies of each of the TFBSs in the heterotypic TFBSs pairs without taking into account their order (Fig. 3f). Across the heterotypic TFBSs pairs, we found that when having two copies of heterotypic TFBSs pairs the orientation for which the expression levels were higher was dependent on the TFBS pair (Fig. 3d, Supplementary Fig. 10). For example, for FOXA1-RXRA and REST-HNF4A, the highest expression was obtained when both TFBSs pairs were found at the non-template orientations (Fig. 3f; t-tests with Bonferroni-corrected p-values). Interestingly, each TFBS orientation that showed the highest expression in heterotypic clusters consistently showed the highest expression in homotypic clusters for that specific TFBS (Figs. 1–2**)**.

We next examined the effect of order for multiple heterotypic TFBF pair copies. We observed that the order between the two heterotypic TFBSs for which expression levels were highest were the same irrespective of the number of consecutive copies of the constituent TFBSs (Fig. 3g, Supplementary Fig. 11). For example, for the heterotypic TFBS pair of HNF1A-CEBPA having the HNF1A TFBS upstream of the CEBPA TFBS resuled in the highest expression irrespective of number of copies (Fig. 3g). These results suggest that the influence of the heterotypic TFBS orientations on regulatory activity are largely driven by the orientation preference of the individual TFBSs.

### Order impacts expression in heterotypic triplet TFBSs

**Fig. 5:**
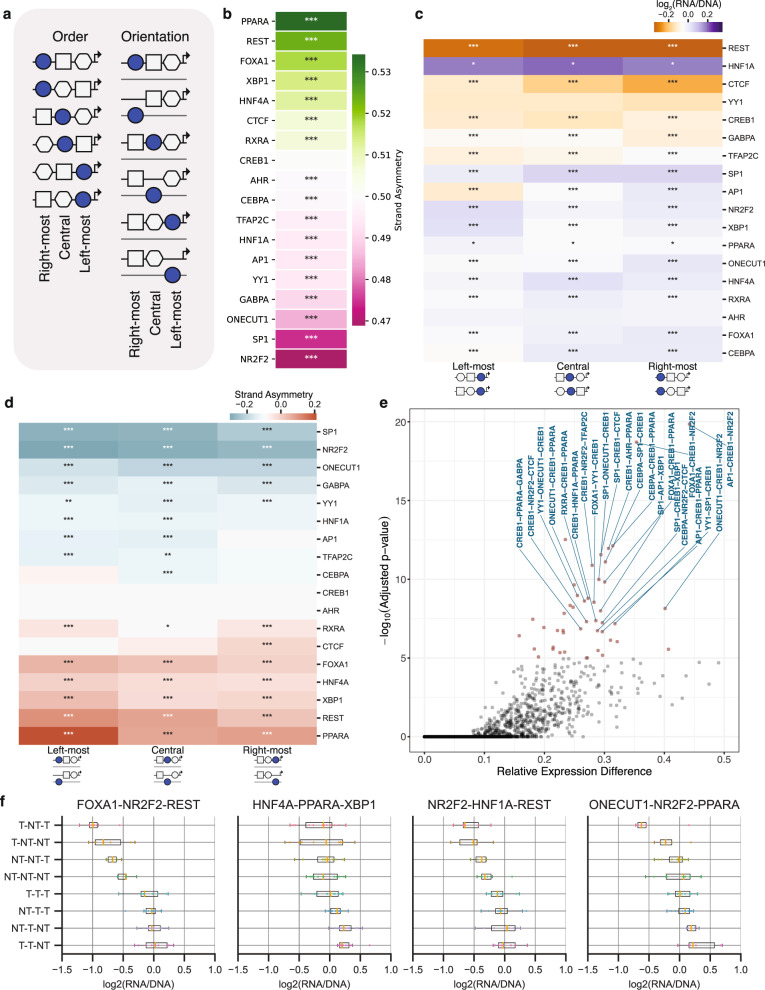
The strand orientation and order of constituent TFBSs in TFBS clusters influences expression levels. **a**, Schematic displaying the positions in which a TFBS can be found within a TFBS triplet, being the rightmost, middle or leftmost TFBS and in two possible orientations. For the TFBS triplet there are eight possible orientations and six different orders. **b**, Strand asymmetry in expression between the template and non-template orientations for each TFBS across the TFBS triplet clusters. Statistical significance was estimated with two-sided t-tests with Bonferroni-corrected p-values. **c**, The order of TFBSs in the TFBS triplet influences expression levels. Across TFBS triplets, we examined if the position of each TFBS influenced expression levels by comparing the expression levels between the occurrences of the TFBS in the left-most, central or right-most positions. Statistical significance was estimated with one-way ANOVA with Bonferroni correction. **d**, Strand asymmetry levels for each of the three positions in the TFBS triplet for each TFBS. Strand asymmetry was calculated here as the difference between the mean expressions for the TFBS at the non-template and template orientations across the TFBS triplets. **e**, The orientation of TFBSs influences expression, estimated with one-way ANOVA with Bonferroni-corrected p-values. **f**, Examples of TFBS triplets with all possible orientations of each TFBS presented. For each TFBS, NT represents non-template and T represents template in the order at which the transcription factor names are listed. Results obtained from *n* = 2 background sequences. Adjusted p-values displayed as * for p-value<0.05, ** for p-value<0.01 and *** for p-value<0.001. In the boxplots, the median is indicated as the center line, the lower and upper limits of the boxplots indicate the first quantile (25^th^ percentile) and the third quantile (75^th^ percentile) respectively, the lower and upper whiskers are the lowest and the maximum value of the data that are within 1.5 times the interquartile range over the 25^th^ and the 75^th^ percentile respectively.

We next analyzed our results for combinations of three heterotypic TFBSs. We first examined whether, similar to heterotypic TFBSs pairs, the order of TFBSs in triplets influences expression levels (Fig. 5a). There are three possible positions for a certain TFBS in heterotypic triplets: 1) the ‘left-most’; 2) ‘central’ or 3) ‘right-most’ position (Fig. 5a). We observed that the position of a TFBS significantly influenced expression for every TFBS except for AHR and YY1. The biggest differences were observed for AP1, CTCF and REST TFBSs (Fig. 5c; one-way ANOVA, Bonferroni correction). For example, for REST we saw a 12.3% difference in expression between the leftmost and rightmost positions. Therefore, the position of TFBSs in TFBS triplets influences expression levels.

### TFBS orientation impacts expression in heterotypic triplets

Three heterotypic TFBSs can be found in eight possible orientations, with each TFBS being in the template or non-template orientations (Fig. 5a). For each TFBS, we investigated if its orientation influenced expression. We found that the orientation of TFBSs for seventeen out of eighteen transcription factors significantly influenced expression, with the exception of the palindromic CREB1 (Fig. 5b; t-test, Bonferroni corrected, p-value<0.05). The expression difference between the two orientations was highest for PPARA and NR2F2 and lowest for AHR.

We next investigated how the strand orientation of TFBSs influences expression in specific TFBS triplets. For each triplet, there were eight possible orientations (two orientations for every TFBS). We examined if there was a significant difference between the orientation of TFBSs and expression levels regarding the orientation of the first, second or third TFBS in the triplet, examined independently. In total, we identified 305 TFBS triplets for which the orientation of the constituent TFBSs significantly influenced expression levels. The majority of the TFBS triplets with significant differences in the groups included the palindromic TFBS of CREB1, which can be explained by the larger number of sequences in the eight groups tested (due to its palindromic nature, CREB1 was double-counted in multiple orientation groups). We had 43 TFBS triplets without CREB1 that had highly statistical differences depending on the orientations. These included for example SP1-AP1-XBP1, FOXA1-NR2F2-REST, HNF4A-PPARA-XBP1 and CEBPA-NR2F2-CTCF1 (Fig. 5e-f; t-test with Bonferroni correction). These results show that in heterotypic TFBS triplet, TFBS orientation can have a significant impact on expression levels.

### TFBS grammar in heterotypic TFBS triplets impacts expression

We next examined if both order and orientation of each TFBS in these heterotypic triplets affects regulatory activity (Fig. 5a). With the exception of CREB1, we found significant differences in the expression between different TFBS orientations for all TFBSs for at least one of the “left-most”, “central” or “right-most” positions, with most TFBSs displaying strand bias in all three positions (Fig. 5d). The strongest biases were observed for NR2F2 and PPARA in the “left-most” positions (Fig. 5d). For example, when NR2F2 was in the “left-most” position there was on average a 1.15-fold higher expression in the template than the non-template orientation. In summary, both order and orientation affect regulatory activity in heterotypic triplets and the orientation effects we observed were largely independent of TFBS order.

### The orientation of TFBSs in TFBS triplets impacts expression

We next investigated how the strand orientation of TFBSs influences expression in specific TFBS triplets. For each triplet, there were eight possible orientations (two orientations for every TFBS). We examined if there was a significant difference between the orientation of TFBSs and expression levels regarding the orientation of the first, second or third TFBS in the triplet, examined independently. In total we identified 305 TFBS triplets for which the orientation of the constituent TFBSs significantly influenced expression levels. The vast majority of the top TFBS triplets included the palindromic TFBS of CREB1, which can be explained by the larger number of sequences in the eight groups tested (because of being palindromic, CREB1 was double-counted in multiple orientation groups). However, we found 43 highly significant TFBS triplets without CREB1 that included SP1-AP1-XBP1, FOXA1-NR2F2-REST, HNF4A-PPARA-XBP1 and CEBPA-NR2F2-CTCF1 among others (Fig. 5e-f; t-test with Bonferroni correction). These results show that in heterotypic triplet TFBSs, the effect of the orientation of non-palindromic TFBSs impacts expression levels across the transcription factors tested and across TFBS clusters.

### TFBS grammar impacts gene expression in the human genome

To further characterize whether similar orientation and order exists in the human genome, we used ChIP-seq data that was available for a subset of transcription factors (CEBPA, CREB1, CTCF, FOXA1, GABPA, HNF1A, HNF4A, JUN, NR2F2, REST, RXRA and YY1). For each of these TFs, we used the most likely ChIP-seq bound TFBSs from the UniBind database^25^. Next, we investigated if the TFBS pairs showed a preference for particular *cis*-regulatory elements. In total, seven groups were examined for their TFBS pair enrichment, namely elements with promoter-like signatures (PLS), promoter-proximal enhancer-like signatures (pELS), distal enhancer-like signatures (dELS), elements with high H3K4me3 and low H3K27ac levels (DNase-H3K4me3), elements with high DNase and CTCF levels (CTCF-only), and elements with high DNase levels but low H3K4me3, H3K27ac, and CTCF levels (DNase-only), or elements with low-DNase levels, previously annotated by the ENCODE Project^26,27^. As expected, we found that most TFBS pairs were depleted in Low-DNase regions. We found that there were clusters of TFBS pairs that were enriched across *cis*-regulatory elements, whereas other TFBS pairs were more enriched at particular *cis*-regulatory elements (Supplementary Fig. 12a).

To further support the importance of TFBS orientation on transcription, we analyzed TFBS orientation in human promoters. We mapped the location of 842 TFBSs across promoters (−2,500 bp to TSS) utilizing position weight matrices from the JASPAR database^20^. We then analyzed strand orientation biases for consecutive homotypic TFBS copies in close proximity to one another (<100 bp) finding that 50.71% of TFBSs analyzed displayed orientation biases, with 49.45% showing same orientation (both template or both non-template) strand preference and only 1.19% showing opposite strand orientation (one template, one non-template) preference for maximum distance of 50 bp between consecutive occurrences (Supplementary Fig. 12b). Among the eighteen transcription factors that we studied, twelve (AP1, CREB1, CTCF, GABPA, NR2F2, ONECUT1, PPARA, REST, RXRA, SP1, TFAP2C, and YY1) showed similar strand orientation biases, with same orientation preference. To take into account potential nucleotide composition biases, we also performed simulations of human promoter sequences controlling for dinucleotide content (see **Methods**) and found the strand asymmetry results to be unchanged.

We also investigated if the order of pairs of TFBS were biased across human promoters. To perform this analysis, we generated all possible TFBS pairs among the 842 TFBSs and examined if the constituent TFBSs were equally likely to be in the most proximal or distal position of the promoter when they co-occurred. We found 97% of TFBS pairs showed a preference in terms of their order for either being proximal or distal (Supplementary Fig. 12c; Binomial test, Bonferroni corrected p-value, p-value<0.05). We also examined the TFBSs within the ChIP-seq peaks from HepG2 data for CEBPA, CTCF, CREB1, FOXA1, GABPA, HNF1A, HNF4A, JUN, NR2F2, REST, RXRA and YY1 transcription factors. We found significant order preference for their TFBS pairs for 13.6% of the pairs (binomial test, p-value<0.05, Bonferroni corrected; Supplementary Fig. 12d). The discrepancy between the predicted motifs and the ChIP-seq bound motifs could be due to repetitive elements or a higher number of unbound TFBSs. Additionally, this observation, i.e., finding that TFBS pair distribution is not random, further supports our MPRA observations that the order of TFBSs in TFBSs pairs influences expression levels.

To provide further support for the functional importance of orientation on gene regulatory activity, we analyzed an MPRA experiment that tested the majority of *cis*-regulatory regions in HepG2 cells (*N* = 164,307), generated as part of the ENCODE project^28^. From the transcription factors present in our library, AP1, CTCF, FOXA1, HNF4A, PPARA, RXRA and YY1 showed significant differences in expression depending on orientation consistent with our MPRA results (Supplementary Fig. 12e**;** t-test, p-value<0.05). We also examined if the order of TFBS pairs of the eighteen TFBSs from our library influenced expression. We found that across the 306 heterotypic TFBS pairs, 29 exhibited significant expression differences depending on their order, primarily for heterotypic pairs involving CTCF, HNF1A and TFAP2C TFBSs (Supplementary Fig. 12f), consistent with our results. We also examined the effect of the position of a TFBS in the MRPA tile across *cis*-regulatory regions in HepG2 cells. We found that six of the studied transcription factors had a statistically significant association between proximity to the TSS and expression levels (CREB1, CTCF, GABPA, HNF1A, HNF4A and NR2F2; Supplementary Fig. 13) and the directionality of the effects were highly consistent across transcription factors with our synthetic MPRA (Supplementary Fig. 4). Next, we examined if the distance between TFBS pairs was biased using predicted TFBSs and ChIP-seq bound TFBSs, using ChIP-seq data from the HepG2 cell line, genome-wide. We observed consistently that there were pairwise distances that were preferred for both the predicted TFBS interactions and for the ChIP-seq bound TFBSs (Supplementary Fig. 14a-b). We replicated these findings in promoter regions, finding that our results were largely unaltered (Supplementary Fig. 14a-b). Taken together, these findings support that the orientation, order and TFBS positioning *cis*-regulatory effects observed in the designed MPRA experiment are also reflected in the human genome.

### TFBS orientation improves predictive models

**Fig. 6:**
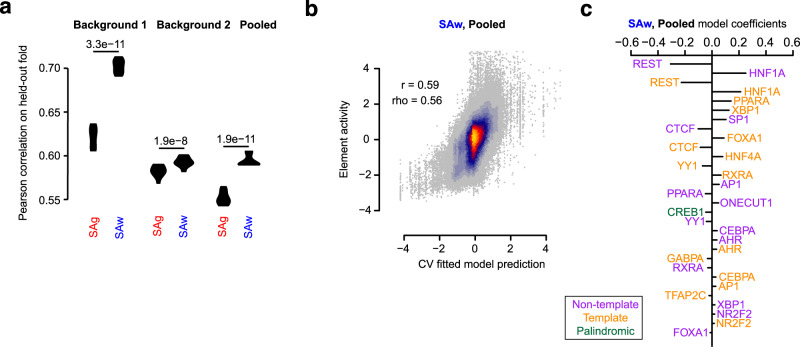
A predictive model that accounts for TFBS orientation provides improved performance. **a**, Violin plot of the Pearson correlation values for the predictive models that are either agnostic (SAg) or aware (SAw) towards TFBS orientation. **b**, Scatter plot of the predictions and observed element activity scores from the pooled data from background sequences 1 and 2. Shown are the final predictions after concatenating the observations for all 10 folds of held-out data. Also indicated are the Pearson (r) and Spearman (rho) correlation values. Regions are colored according to the density of data from light blue (low density) to yellow (high density). **c**, The top thirty coefficients from the strand-aware lasso regression model trained on the full pooled dataset. Non-template and template orientations have been colored purple and orange, and their performance has been examined independently. Palindromic motifs are depicted in green.

Sequence based models provide a powerful approach to predict regulatory activity^−^^29–31^. We trained a lasso regression model that predicted sequence activity based on 36 features, which were the TFBSs of the eighteen transcription factors in the two orientations, using tenfold cross-validation. The performance of the model utilizing either the first or second background sequence had correlation coefficients (r) of 0.70 and 0.59 respectively (Fig. 6a-b, Supplementary Fig. 15). The lasso regression model that took into account the orientation of TFBSs performed 19.34% and 2.13% better than the model that did not consider the orientations (p-values<0.001 in all cases). The discrepancy between the two background templates can be explained by the lower and more variable expression of the second background template, resulting in smaller improvements in the performance. The combined model (Pooled), which was trained using sequences from both background templates, improved performance by 7.7% with the incorporation of TFBS orientation. Interestingly, the lasso coefficients of the TFBSs were in many cases different in magnitude between the two orientations and even had opposite directionality, most notably for PPARA, FOXA1 and RXRA, consistent with our observations finding differences in their expression dependent on orientation (Fig. 6c).

## Discussion

This study investigated the *cis*-regulatory syntax and the influence of TFBS orientation and order on transcription utilizing an MPRA that tested 209,440 sequences encompassing all possible pair and triplet combinations, permutations and orientations of eighteen liver-associated TFBSs. We found that TFBS orientation and order have a major effect on gene regulatory activity, which was validated by analyzing human promoters and MPRA data from HepG2 cells. We then used orientation as a variable to augment a predictive model, showing that it significantly improves performance by up to 19%.

**Fig. 7:**
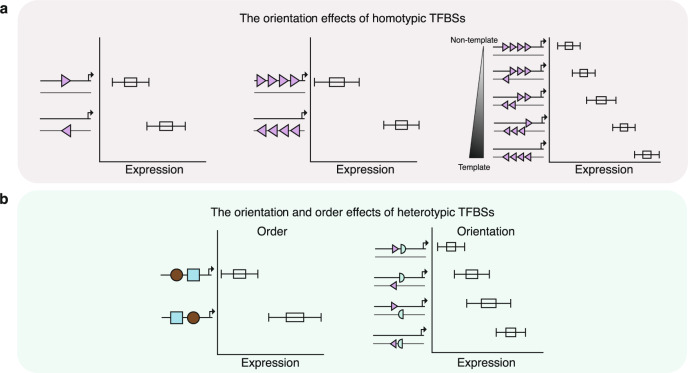
Schematic illustration of the learned *cis*-regulatory syntax. **a**, The orientation of homotypic TFBSs influences expression levels (left panel) and the magnitude of the effect is proportional to the number of copies (middle panel). The proportion of TFBS copies in each orientation is associated with expression levels (right panel). **b**, The order of heterotypic TFBSs and their orientation influence expression levels. Multiple consecutive copies of heterotypic TFBSs show larger differences.

This study provides several important observations on regulatory grammar. These include: 1) TFBS orientation has a major effect on regulatory activity (Table 1); 2) The effects of TFBS orientation increase with added copies of homotypic TFBS (Table 1); 3) Orientation has a major impact on regulatory activity in heterotypic TFBSs (Table 2); 4) The order with which heterotypic TFBSs are placed can significantly influence expression levels (Fig. 7; Table 2).

**Table 1:** Summary of the main *cis*-regulatory grammar findings for each TFBS in homotypic and heterotypic clusters

Homotypic	
Copy Number	Upregulate: AP1, CEBPA, FOXA1, HNF1A, HNF4A, PPARA, SP1, XBP1; Downregulate: CREB1, CTCF, GABPA, REST, TFAP2C, ΥΥ1
Orientation	AHR, FOXA1, HNF4A, NR2F2, ONECUT1, PPARA, REST, RXRA, SP1, TFAP2C, XBP1, YY1
Orientation +Copy Number	Consistent expression levels across copy number and orientations:HNF1A, AP1, CTCF, GABPA, YY1, REST; Consistent expression levels across copy number and one orientations: AHR, CEBPA, TFAP2C, HNF4A, PPARA; Antithetical expression levels across copy number and orientations: ONECUT1, RXRA, FOXA1, XBP1
Distance from TSS	Upregulate: AHR, CREB1, CTCF, GABPA, HNF1A, REST, SP1, XBP1, YY1; Downregulate: FOXA1 and ONECUT1
Distance from TSS + Orientation	Consistent expression levels across distance and orientations: CEBPA, AHR, HNF4A, PPARA, REST, XBP1; Consistent expression levels across distance and one orientations: CTCF, HNF1A, AP1,GABPA,TFAP2C,SP1,ONECUT1,RXRA,FOXA1; Antithetical expression levels across distance and orientations: None

**Table 2:** Summary of the main *cis*-regulatory grammar findings for each TFBS in heterotypic clusters

Heterotypic	
Order	Significant bias for proximal position: YY1, TFAP2C, ONECUT1, SP1, CREB1, AP1, FOXA1, CEBPA
	Significant bias for distal position: RSRA, REST, HNF1A, XBP1,GABPA, NR2F2, CTCF, AHR, PPARA
Orientation	Significant strand biases in heterotypic pairs: PPARA, HNF4A, RXRA, REST, CEBPA, GABPA, AHR, HNF1A, YY1, AP1, TFAP2C, FOXA1, CTCF, XBP1, NR2F2, SP1, ONECUT1
	Significant strand biases in heterotypic triplets: PPARA, REST, FOXA1, XBP1, HNF4A, CTCF, RXRA, AHR, CEBPA, TFAP2C, HNF1A, AP1, YY1, GABPA, ONECUT1, SP1, NR2F2
Triplet order	Significant order biases in heterotypic triplets: REST, HNF1A, CTCF, CREB1, GABPA, TFAP2C, SP1, AP1, NR2F2, XBP1, PPARA, ONECUT1, HNF4A, RXRA, FOXA1, CEBPA
Triplets orientation + Order	Significant order and strand biases in heterotypic triplets: SP1, NR2F2, ONECUT1, GABPA, YY1, HNF1A, AP1, TFAP2C, CEBPA, RXRA, CTCF, FOXA1, HNF4A, XBP1, REST, PPARA

Previous work has examined the effect of genomic element orientation on expression in both single locus experimental studies^7,32^ and via high-throughput assays^9,13,18,33^. For example, using STARR-seq, it was shown that orientation of Alu repeat elements and DNA octamers are associated with expression differences^18^. A previous MPRA identified that the orientation of the tested tiles influenced expression^17^, while in another MPRA experiment statistically significant differences in expression relative to the orientation of a set of core-promoter TFBS were shown^13^. However, to our knowledge this is the first study that examined TFBS orientation at this scale, showing that it has such a significant effect. For certain TFBS, such as RXRA, in homotypic TFBS clusters the expression levels increase analogously to the proportion of TFBSs in the same orientation (Fig. 1e-f). However, this rule is dependent on the studied TFBS, with other TFBSs such as YY1 showing highest expression with a balanced proportion of template and non-template copies in homotypic TFBS clusters (Fig. 2g). For XBP1, the number of homotypic TFBS copies in the non-template orientation led to increased expression, whereas multiple copies in the template orientation led reduced expression (Fig. 1f), further reinforcing the notion that orientation is of primary importance in understanding the *cis*-regulatory code. We also used the palindromic TFBS CREB1 as a negative control, and did not observe any effects of orientation with this TFBS, further corroborating our results. In general, our findings are in accordance with experimental work indicating that the orientation of transcription factor binding impacts transcription factor binding and transcriptional potency^32,34,35^. Here, we identify rules that dictate these effects, which can be further learned and incorporated via machine learning models.

While we saw that order had a significant effect on regulatory activity for heterotypic TFBS pairs and triplets, this was not the case across all TFBSs. For example, CREB1, HNF1A and XBP1 showed highly significant differences if they were in proximal or distal positions (Fig. 3g). These results were consistent when placing multiple consecutive copies of the two heterotypic TFBSs, albeit the magnitude of the effect of order was amplified with consecutive copies of each TFBS (Fig. 3g). We also observed that the above results are dependent on the TFBS studied, indicating that a complete understanding of these results would require examining additional TFBSs.

Previous work using CAP-SELEX experiments found the orientation and order of heterotypic TFBS pairs significantly influences transcription factor binding^9^. For heterotypic transcription factor pairs, the orientation of the constituent TFBSs evidently influences expression levels for many TFBSs to the extent that occurrences of TFBSs of the same transcription factor in opposite orientations do not cluster together for certain TFBSs (Fig. 3b). For heterotypic transcription factor pairs with multiple inserted copies of each TFBS in the MPRA tile, there is a preferred orientation for both members of the pair and expression levels increase relative to the proportion of consecutive TFBS copies in the optimal orientation (Fig. 7b).

There are several limitations of this study. Our study utilized a liver cell line, HepG2, as a small number of transcription factors are thought to be involved in its regulation^36^. For future studies it would be of interesting to investigate many additional cell types with more complex transcription factor regulation. The examination of additional kmers for each TF in future studies can enable the examination of the extent of consistency of the findings across different binding sites. It is important to also note that due to the degeneracy of the *cis*-regulatory code, other TFs could potentially bind to the sequences of the studied TFBSs with lower affinity. Also, we note that the results obtained for the expression differences by altering the order of TFBSs could be influenced by the distance of the constituent TFBSs from the TSS. Furthermore, future work will be required to provide a granular investigation with base-pair resolution to examine TFBS interactions within the TFBS clusters for a range of inter-motif distances, to further understand the effects of DNA structural parameters such as the helical turn. It will also be interesting to test cells across differentiation time points and other perturbations. Despite testing over 200,000 sequences, we were limited in the number of TFBS combinations we could test and also only had two background sequences. Also, we note that the correlation between the replicates ranging between 0.74 and 0.79 is not as high as previous smaller lentiMPRA libraries generated by our laboratory^22,37^, and is likely due to the large size of the library (*N* = 209,440 sequences). While we observed correlation between the two background sequences, the second construct had a low activity, below the negative control, resulting in higher noise to signal ratio. It is also important to note that while MPRAs provide the ability to test hundreds of thousands of sequences for their regulatory activity, they are an artificial system, testing sequences out of their genomic context. While our genomic analyses validate several of our findings, future work involving high-throughput mutagenesis in the endogenous locus could provide results in the natural context of these sequences.

Our findings have important implications across multiple genomic disciplines. Firstly, incorporation of orientation in predictive models result in improved performance, indicating that the orientation of TFBSs is part of the *cis*-regulatory grammar. Secondly, improved understanding of regulatory element variation can lead to improved comprehension of disease-associated variants. Third, advances in our understanding of the regulatory code could enable improved design of synthetic sequences for therapeutic targets. Additionally, incorporation of orientation and order effects in evolutionary analyses could result in a better understanding of the evolution or generation of *cis*-regulatory sequences such as enhancers and promoters.

## Methods

### HepG2 cell culture

HepG2 cells were purchased from Cell and Genome Engineering Core at UCSF (STR profile validated) and were tested negative for mycoplasma using Universal Mycoplasma Detection Kit (30-1012 K, American Type Culture Collection). Cells were maintained in EMEM (ATAC; 302003) supplemented with 10% FBS (VWR; 89510-194) and cultured on collagen coated surface (Sigma; 125-50). Cells were passaged with TrypLE (ThermoFisher; 12604039,) until reaching 80% confluence.

### Luciferase assay

Two template sequences with 168 bp and 200 bp length, as well as a negative control sequence were synthesized by Twist Biosciences. Each sequence includes flanking sequences that are homologous to the insertion site of the lentiMPRA vector and MPRA adaptor sequences on both sides, so as to generate the same vector context as the lentiMPRA library. The respective nucleotide sequences and genetic coordinates are shown in Supplementary Table 2. Synthesized sequences were amplified by PCR using forward and reverse primers (F, CTCACTCAGCCTGCATTTCTG; R, GCTTCCATTATATACCCTCTAGTG), and inserted into *Xba*I and *Sbf*I site of pLS-mP-Luc (Addgene, #106253). In addition, as a positive control, The SV40 sequence is also amplified from pLS-SV40-mP-EGFP (Addgene, #137724) and cloned into pLS-mP-Luc. Each of the plasmids were packaged into lentivirus together with pLS-S40-mP-Rluc (Addgene, #106292) using Lenti-Pac HIV Expression Packaging Kit (Genecopoeia, LT002), according to the manufacturer’s instructions. The molarity ratio of the firefly and renilla plasmids was 2:1. The lentivirus were concentrated using the Lenti-X concentrator (Takara, 631232). 1×10^4^ HepG2 cells/well were seeded in a 96-well plate, cultured for 24 hours, and infected with the lentivirus. Three independent replicate cultures were infected. After 2 days, firefly and renilla luciferase activities were measured on a GloMax Explorer (Promega) using the Dual-Luciferase Reporter Assay System (Promega).

### lentiMPRA library design

Two neutral genomic constructs, each 200 bp in length (Supplementary Fig. 1a), were used for the generation of the MPRA library. The MPRA library was designed to include TFBSs for 18 transcription factors and for each transcription factor a single TFBS kmer was used (Supplementary Table 1). For homotypic TFBSs, one, two, three, four, five, six or eight copies of the TFBS kmer were inserted in the non-template or template orientation. For heterotypic TFBS pairs, all combinations were tested with one, two or three copies of each TFBS kmer of the TFBS pair were inserted in the non-template or template orientation. For heterotypic TFBS triplets, all combinations were generated with each TFBS being either on the non-template or the template orientation. For placing multiple TFBSs within the MPRA tiles, three distances were used, 5 bp, 10 bp or the most frequent genomic distance between each pair of TFBSs with the exception of TFBS triplets in which only the most frequent genomic distance was used due to exceedingly large numbers.

For the eighteen transcription factors in the MPRA, for identifying the optimal distance between them, the following sequences were used: for AP1 the motif TGACTCA, for CREB1 the motif TGACGTCA and the PWMs with the following JASPAR IDs: “MA0102.3” (CEBPA), “MA0139.1” (CTCF), “MA0148.3” (FOXA1), “MA0062.2” (GABPA), “MA0046.2” (HNF1A), “MA0114.3” (HNF4A), “MA1111.1” (NR2F2), “MA0679.1” (ONECUT1), “MA1148.1” (PPARA_RXRA), “MA0138.2” (REST), “MA0512.2” (RXRA), “MA0079.3” (SP1), “MA0524.2” (TFAP2C), “MA0844.1” (XBP1), “MA0095.2” (YY1) and “MA0148.3” (FOXA1). For AHR, the HOCOMOCO PWM was used: “AHR_HUMAN.H11MO.0 ”.

### lentiMPRA library construction and association sequencing

lentiMPRA plasmid library was generated as previously described in^23^ with minor modifications. Briefly, designed oligo pool was synthesized (Agilent) and resuspended in Elution buffer (19086, Qiagen) to 25 nM. Two rounds of PCR were performed on 12 μl oligos using NEBNext UltraII Q5 master mix (M0544L, NEB) and two primer sets (5BC-AG-f01/r01 and 5BC-AG-f02/r02). The following cycling program was used: 98 °C for 30 s, 5 or 12 cycles of 98°C for 10 s, 67°C for 20 s, 72°C for 20 s, and a final extension at 72°C for 2 min. PCR reaction was cleaned up using SPRIbeads (B23318, Beckman Coulter). After amplification, a minimal promoter, barcodes, and overlapping ends for recombination were added to each oligo. PCR amplicons were cloned into linearized (AgeI/SbfI) lentiviral vector pLS-SceI (Addgene, 137725) using NEBuilder HiFi DNA Assembly master mix (E2621S, NEB) and empty vectors were removed by I-SceI (R0694S, NEB) digestion. Recombination products were transformed to electrocompetent cells (C3020K, NEB) following manufacture’s protocol and incubated overnight at 37°C on 15 cm LB agar plates with 100 μl of 100 mg/ml carbenicillin (10177012, Gibco). Roughly 10 million colonies were harvested using the Plasmid Plus Midi Kit (12945, Qiagen) so that each oligo has ~50 barcodes on average. To identify the complete list of barcodes associated with each oligo, a sequencing library was prepared from 500 ng plasmid library by PCR amplification (primer set: P5-pLSmP-ass-i# / P7-pLSmP-ass-gfp, cycling program: 98°C for 30 s; 7 cycles of 98°C for 10 s, 65°C for 75 s; a final extension at 65°C for 5 min) and sequenced on Illumina NextSeq Mid-output with custom primers (Read1: pLSmP-ass-seq-R1, Read2: pLSmP-ass-seq-R2, i7 index: pLSmP-ass-seq-ind1, i5 index: pLSmP-rand-ind2) using the following setting: 146 + 146 + 15 + 10 bp (Read1 + Read2 + i7 index + i5 index).

### Lentivirus packaging, titration and infection

Lentivirus was produced by co-transfecting lentiMPRA plasmid library and helper plasmids into 15 cm dishes of 293 T cells using Lenti-Pac HIV expression packaging kit (GeneCopoeia; LT002,). After 48 h of transfection, crude solution was harvested and filtered through a 0.45 μm PES filter unit to remove cell debris. Lentivirus was concentrated 100 times using Lenti-X concentrator (Takara; 631232) and kept at 4 °C for less than 3 weeks. Titration was performed as previously described^23^. 4 ml of 1.2E + 05 TU/μl lentivirus was added to 6 million HepG2 in a 15 cm dish per replicate to achieve an MOI of 50 and roughly 30 integrations per barcode. Three replicates were performed. Polybrene (MilliporeSigma; TR1003G) was added to culture medium to a final concentration of 8 μg/ml to improve transduction efficiency. A medium change without polybrene was performed on the next day.

### DNA/RNA extraction and sequencing

Three days after lentivirus infection, DNA and RNA were extracted using the Allprep mini kit (Qiagen; 80204). In brief, each biological replicate was rinsed with DPBS then lysed in 2.4 ml RLT Plus buffer with 1% 2-mercaptoethanol and homogenized in QIAshredder (Qiagen; 79656) columns via a 2 min full speed centrifuge. Four DNA columns and eight RNA columns were used. ~800 μl of 40 ng/μl gDNA and ~400 μl of 100 μg/μl RNA were harvested per replicate. The number of viral integrations per cell was measured by qPCR and the average MOI of three replicates was 55. To prepare sequencing libraries, 5 μg RNA was used for reverse transcription to generate cDNA using Superscript IV RT (Invitrogen; 18090200) and a custom primer P7-pLSmp-ass16UMI-gfp. cDNA and 16 μg gDNA were amplified separately for three cycles (primer: P7-pLSmP-ass16UMI-gfp/P5-pLSmP-5bc-i#; cycling program: 98 °C, 10 s, 72 °C, 35 s; a final extension at 72 °C for 2 min) and cleaned up using SPRIbeads at ×1.4 ratio. 16 bp barcodes in the DNA template were amplified and added with a unique molecular identifier (UMI), an index and Illumina P5/P7 sequence. A qPCR was then performed (primer: P5/P7) on 1/10 of first PCR amplicons to determine the number of cycles needed for the second PCR (Ct value when ΔRn = ~2 million). After 10 cycles of second PCR, final products were purified using x1.2 SPRIbeads. DNA and RNA barcode libraries were pooled in 1:3 mole ratio and sequenced with 2 runs of NextSeq High-output with custom primers (Read1: pLSmP-ass-seq-ind1, Read2: pLSmP-bc-seq, i7 index: pLSmP-UMI-seq, i5 index: pLSmP-5bc-seq-R2) using the following setting: 15 + 15 + 16 + 10 bp (Read1 + Read2 + i7 index + i5 index).

### Analysis of lentiMPRA experiment

MPRA computational analysis was performed with algorithms adjusted from MPRAflow^23^. For barcode insert mapping and filtering, we called a consensus sequence from the paired-end reads associating with barcode sequence from the index read. We aligned all consensus sequences back to all designed sequences (inserts) using BWA MEM (version 0.7.17-r1188)^38^. We used the NM tag with up to one mismatch as a strict filter. For RNA/DNA barcode counting and ratio normalization, RNA and DNA barcodes for each of the three replicates were sequenced on an Illumina NextSeq instrument, and UMI was used to remove PCR duplicates and the inserts with associated barcode counts lower than 3 are removed. For analyses involving the two background sequences, unless otherwise stated the results were combined. For analysis of homotypic TFBS kmers, sequences with other TFBS kmers were excluded; for heterotypic pairs, sequences including TFBSs different than the TFBS kmers of the pair were excluded and for triplets sequences with TFBSs different than the TFBS kmers of the triplet were excluded. Non-template to template expression difference was estimated as the difference in mean expression between sequences with homotypic TFBSs in the non-template versus the template orientation and statistical significance was estimated with t-tests and Bonferroni correction. Relative expression difference was calculated as the absolute value of the mean expression difference for sequences found in two different orientations.

### Human promoter analysis

Gene annotation from GENCODE v40 was used throughout the study^39^. Promoters were defined from the reference gene GTF file as −2500 bp upstream of the start of the gene to its start. Identification of TFBSs at promoters was performed with BEDTools intersect function. RNA-seq data from HepG2 were derived from the Roadmap Epigenomics Consortium^40^ using the processed FPKM expression matrix.

### Transcription factor binding site maps

Position frequency matrices (PFMs) of transcription factors were derived from JASPAR (release 2022) for the non-redundant CORE vertebrate collection (http://jaspar.genereg.net/download/CORE/JASPAR2022_CORE_vertebrates_non-redundant_pfms_meme.zip)^20^ and motif scanning was performed with FIMO^41^ using as background model the nucleotide frequencies across the human genome and requiring a minimum p-value <10^−4^. Simulations of the human genome were generated using uShuffle, controlling for dinucleotide content^42–45^. Consecutive homotypic TFBSs with inter-motif distance <100 bp were analyzed across the human genome and within promoter sequences for orientation strand preference with the null hypothesis being that consecutive TFBSs are equally likely to be on the same or opposite orientation. Strand asymmetry was defined as the number of consecutive TFBSs in the same orientation over the total number of consecutive TFBSs in either orientation and statistical significance was estimated with a binomial test and Bonferroni-corrected p-values.

### TFBS pair enrichment across *cis*-regulatory elements

ChIP-seq bound TFBS were derived from UniBind^25^ for CTCF (“ENCSR000AMA”), CREB1 (“ENCSR000BVL”), FOXA1 (“ENCSR000BLE”), GABPA (“ENCSR000BJK”), HNF1A “ENCSR800QIT, HNF4A (“ENCSR000BLF”), JUN (“ENCSR000EEK”), NR2F2 (“ENCSR000BVM”), REST (“ENCSR000BOT”), RXRA (“ENCSR000BHU”) and YY1 (“ENCSR000BNT”). Putative *cis*-regulatory elements for HepG2 were derived from^26^. Enrichment at each *cis*-regulatory element for each TFBS pair was measured as the number of TFBS pairs within 100 bp from each other overlapping that element over the total number of TFBS pairs within 100 bp across all genomic elements.

### HepG2 ENCODE MPRA library analyses

A lentiMPRA dataset that has been generated by our group as part of the ENCODE consortium (Accession: “ENCSR359FTN”) of 164,307 sequences tested was analyzed. TFBSs were detected with FIMO^41^ as described in the previous section and the orientation was measured from the orientation of the tiled sequence relative to the construct’s promoter orientation. The effect of the orientation (template/non-template) and the order were tested with Mann-Whitney U tests for sequences with the TFBS in each of the two orientations or for TFBS pairs in each of the two orders respectively.

### Predictive modeling with lasso regression

A lasso regression model was trained as described previously^17,22^ and tested on held-out data using a 10-fold cross-validation procedure. The model was either trained using 18 features, each representing the number of occurrences of each TFBS in each sequence or 36 features, taking also the orientation at which each TFBS was found. The top 30 coefficients with highest magnitude were calculated and shown.

### Reporting summary

Further information on research design is available in the Nature Portfolio Reporting Summary linked to this article.

## Supplementary information

Supplementary information

Additional Supplementary Files

Supplementary Data 1

Reporting Summary

## Data Availability

The lentiMPRA data generated in this study have been deposited in the GEO database under accession code “PRJNA854975”. Source data are provided with this paper.

## Source data

Source Data

## Code availability

All code packages and pipelines are publicly available. The GitHub link is at: https://github.com/IliasGeoSo/TFBSs_grammar

## References

[1] Chatterjee S, Ahituv N (2017). Gene regulatory elements, major drivers of human disease. Annu. Rev. Genomics Hum. Genet..

[2] Manolio TA (2009). Finding the missing heritability of complex diseases. Nature.

[3] Maurano, M. T. et al. Systematic localization of common disease-associated variation in regulatory DNA. *Science* **337**, 1190–1195 (2012). 10.1126/science.1222794 PMC3771521 22955828

[4] Shendure J, Akey JM (2015). The origins, determinants, and consequences of human mutations. Science.

[5] Matharu N, Ahituv N (2020). Modulating gene regulation to treat genetic disorders. Nat. Rev. Drug Discov..

[6] Spitz F, Furlong EEM (2012). Transcription factors: from enhancer binding to developmental control. Nat. Rev. Genet..

[7] Panne D (2008). The enhanceosome. Curr. Opin. Struct. Biol..

[8] Kulkarni MM, Arnosti DN (2003). Information display by transcriptional enhancers. Development.

[9] Jolma A (2015). DNA-dependent formation of transcription factor pairs alters their binding specificity. Nature.

[10] Georgakopoulos-Soares I (2021). Asymmetron: a toolkit for the identification of strand asymmetry patterns in biological sequences. Nucleic Acids Res..

[11] Zhang L, Guarente L (1994). The yeast activator HAP1–a GAL4 family member–binds DNA in a directly repeated orientation. Genes Dev..

[12] King DA, Zhang L, Guarente L, Marmorstein R (1999). Structure of a HAP1-DNA complex reveals dramatically asymmetric DNA binding by a homodimeric protein. Nat. Struct. Biol..

[13] Weingarten-Gabbay S (2019). Systematic interrogation of human promoters. Genome Res..

[14] Tippens ND (2020). Transcription imparts architecture, function and logic to enhancer units. Nat. Genet..

[15] Avsec Ž (2021). Base-resolution models of transcription-factor binding reveal soft motif syntax. Nat. Genet..

[16] Inoue F, Ahituv N (2015). Decoding enhancers using massively parallel reporter assays. Genomics.

[17] Klein JC (2020). A systematic evaluation of the design and context dependencies of massively parallel reporter assays. Nat. Methods.

[18] Roberts BS (2021). Genome-wide strand asymmetry in massively parallel reporter activity favors genic strands. Genome Res..

[19] Smith RP (2013). Massively parallel decoding of mammalian regulatory sequences supports a flexible organizational model. Nat. Genet..

[20] Castro-Mondragon JA (2022). JASPAR 2022: the 9th release of the open-access database of transcription factor binding profiles. Nucleic Acids Res..

[21] Kulakovskiy IV (2018). HOCOMOCO: towards a complete collection of transcription factor binding models for human and mouse via large-scale ChIP-Seq analysis. Nucleic Acids Res..

[22] Inoue F (2017). A systematic comparison reveals substantial differences in chromosomal versus episomal encoding of enhancer activity. Genome Res..

[23] Gordon MG (2020). lentiMPRA and MPRAflow for high-throughput functional characterization of gene regulatory elements. Nat. Protoc..

[24] Chong JA (1995). REST: A mammalian silencer protein that restricts sodium channel gene expression to neurons. Cell.

[25] Puig RR, Boddie P, Khan A, Castro-Mondragon JA, Mathelier A (2021). UniBind: maps of high-confidence direct TF-DNA interactions across nine species. BMC Genomics.

[26] ENCODE Project Consortium (2020). Expanded encyclopaedias of DNA elements in the human and mouse genomes. Nature.

[27] ENCODE Project Consortium. An integrated encyclopedia of DNA elements in the human genome. *Nature* **489**, 57–74 (2012). 10.1038/nature11247 PMC3439153 22955616

[28] Agarwal, V. et al. Massively parallel characterization of transcriptional regulatory elements in three diverse human cell types. *bioRxiv* 2023.03.05.531189. 10.1101/2023.03.05.531189.

[29] Agarwal V, Shendure J (2020). Predicting mRNA abundance directly from genomic sequence using deep convolutional neural networks. Cell Rep..

[30] Zhou J (2018). Deep learning sequence-based ab initio prediction of variant effects on expression and disease risk. Nat. Genet..

[31] Avsec Ž (2021). Effective gene expression prediction from sequence by integrating long-range interactions. Nat. Methods.

[32] Natesan S, Gilman MZ (1993). DNA bending and orientation-dependent function of YY1 in the c-fos promoter. Genes Dev..

[33] Grossman SR (2017). Systematic dissection of genomic features determining transcription factor binding and enhancer function. Proc. Natl Acad. Sci. USA.

[34] Chytil, M., Peterson, B. R., Erlanson, D. A. & Verdine, G. L. The orientation of the AP-1 heterodimer on DNA strongly affects transcriptional potency. *Proc. Natl Acad. Sci.* **95** 14076–14081 (1998). 10.1073/pnas.95.24.14076 PMC24329 9826656

[35] Falvo JV, Parekh BS, Lin CH, Fraenkel E, Maniatis T (2000). Assembly of a functional beta interferon enhanceosome is dependent on ATF-2–c-jun heterodimer orientation. Mol. Cell. Biol..

[36] Krivan W, Wasserman WW (2001). A predictive model for regulatory sequences directing liver-specific transcription. Genome Res..

[37] Inoue F, Kreimer A, Ashuach T, Ahituv N, Yosef N (2019). Identification and massively parallel characterization of regulatory elements driving neural induction. Cell Stem Cell.

[38] Li H, Durbin R (2009). Fast and accurate short read alignment with Burrows-Wheeler transform. Bioinformatics.

[39] Frankish A (2021). GENCODE 2021. Nucleic Acids Res..

[40] Roadmap Epigenomics Consortium (2015). Integrative analysis of 111 reference human epigenomes. Nature.

[41] Grant, C. E., Bailey, T. L. & Noble, W. S. FIMO: scanning for occurrences of a given motif. *Bioinformatics* **27**, 1017–1018 (2011). 10.1093/bioinformatics/btr064 PMC3065696 21330290

[42] Jiang M, Anderson J, Gillespie J, Mayne M (2008). uShuffle: a useful tool for shuffling biological sequences while preserving the k-let counts. BMC Bioinf..

[43] Glover JN, Harrison SC (1995). Crystal structure of the heterodimeric bZIP transcription factor c-Fos-c-Jun bound to DNA. Nature.

[44] Carlezon WA, Duman RS, Nestler EJ (2005). The many faces of CREB. Trends Neurosci..

[45] Fraser JD, Martinez V, Straney R, Briggs MR (1998). DNA binding and transcription activation specificity of hepatocyte nuclear factor 4. Nucleic Acids Res..

