# Supplementary material for: Transcription factor binding site orientation and order are major drivers of gene regulatory activity
Source: Nat Commun. 2023 Apr 22;14:2333. doi: 10.1038/s41467-023-37960-5 (PMC10122648; doi:10.1038/s41467-023-37960-5)
Supplement: Supplementary file 1 — Supplementary information [file 41467_2023_37960_MOESM1_ESM.pdf]

Supplementary Information

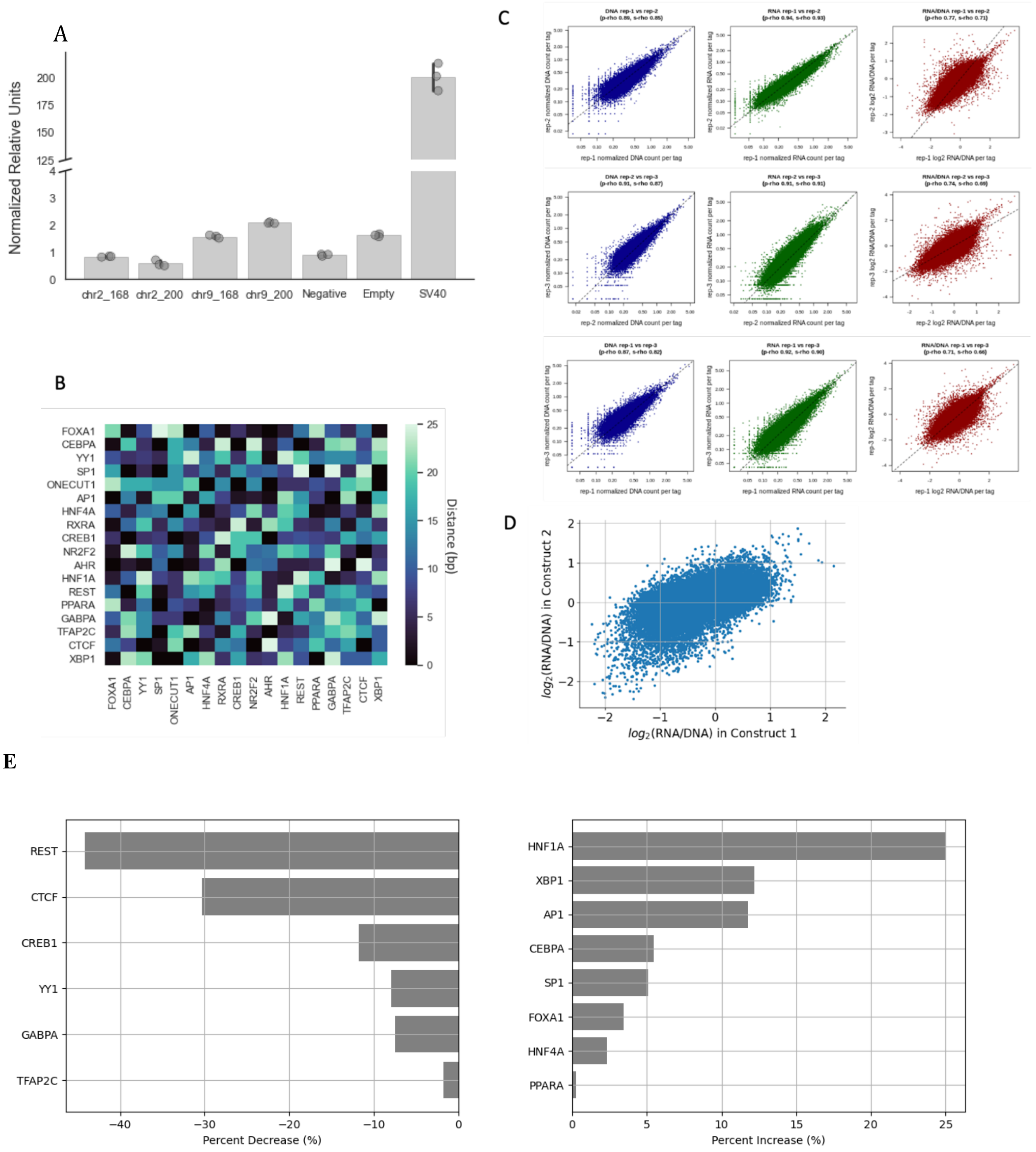

Supplementary Figure 1. a, Luciferase expression of each construct tested. Data are represented as mean values with error bars representing standard deviation. **b,** Correlation between biological replicates for DNA, RNA and RNA/DNA barcodes recovered for each sequence. **c,** Most frequent genomic distance used for every TFBS pair. **d,** Scatter-plot showing the association between the expression levels of each sequence in the two constructs. **e,** Percent change between a single copy and four or more copies of each TFBS.

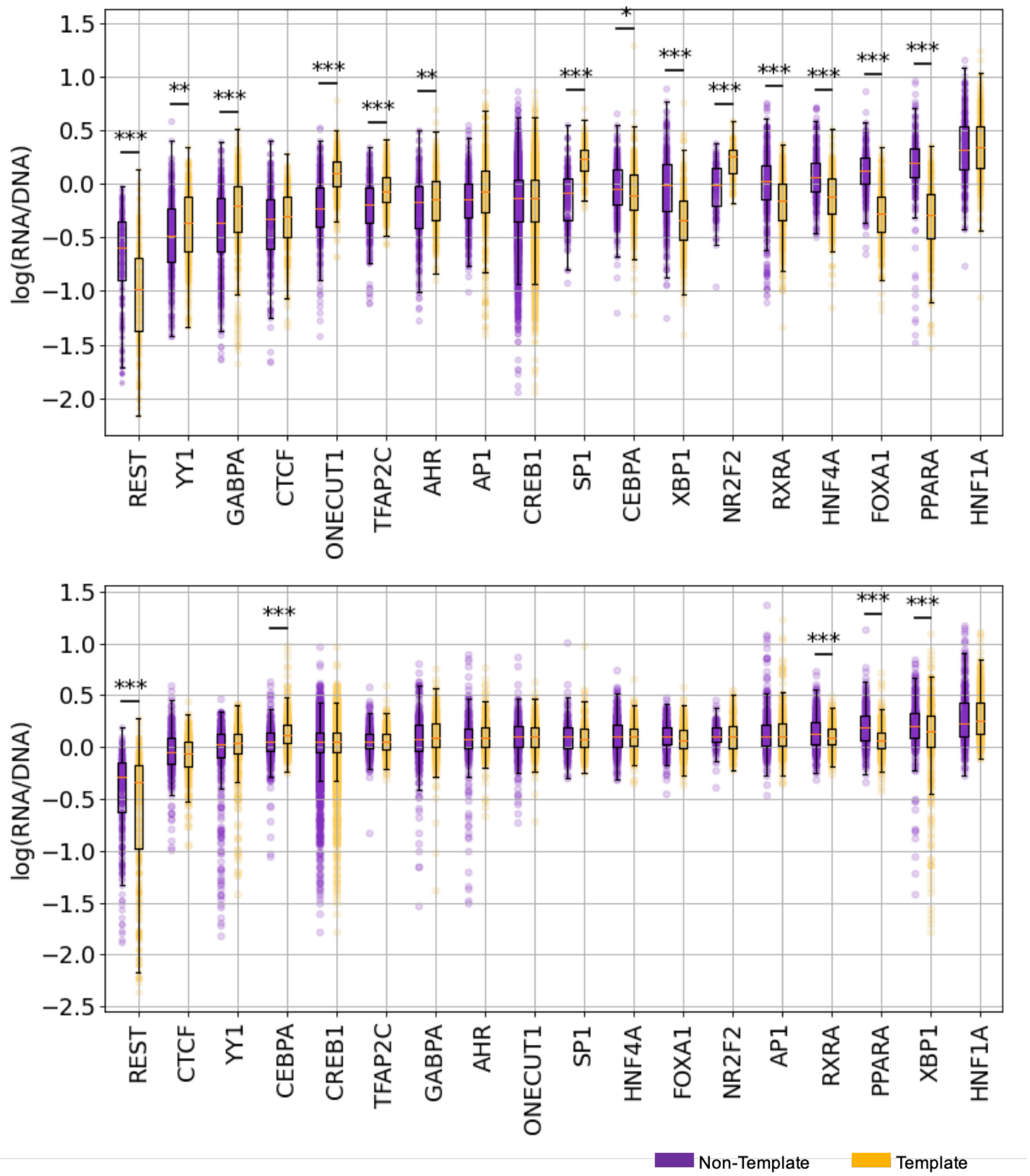

Supplementary Figure 2. Expression levels for sequences with one or more occurrences of a TFBS at the template or the non-template orientation in yellow and purple respectively using background sequence (top) or background sequence two (bottom). Strand asymmetry was calculated as the ratio of the mean expression for sequences with the TFBSs over the mean expression for sequences at booth orientations. Statistical significance calculated with t-test and Bonferroni-corrected p-values. Results obtained from n=1 background sequences. In the boxplots, the median is indicated as the center line, the lower and upper limits of the boxplots indicate the first quantile (25th percentile) and the third quantile (75th percentile) respectively, the lower and upper whiskers are the lowest and the maximum value of the data that are within 1.5 times the interquartile range over the 25th and the 75th percentile respectively.

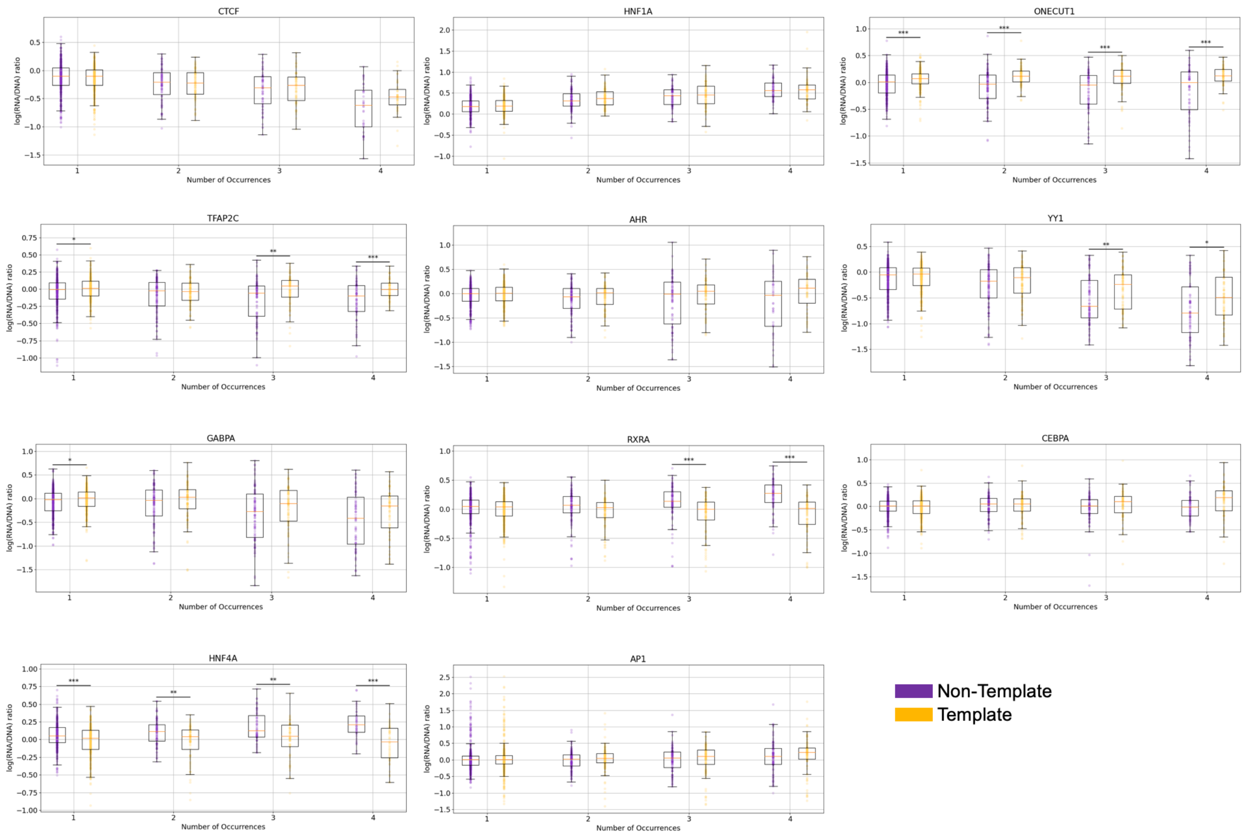

Supplementary Figure 3. Association between number of copies of TFBSs in the MPRA and expression levels. The template (yellow) and non-template (purple) orientations are shown separately for each TFBS. Statistical significance was estimated with t-test with Bonferroni-corrected p-values. For transcription factors SP1 and NR2F2 we did not recover sufficient barcodes. Results obtained from n=2 background sequences. In the boxplots, the median is indicated as the center line, the lower and upper limits of the boxplots indicate the first quantile (25th percentile) and the third quantile (75th percentile) respectively, the lower and upper whiskers are the lowest and the maximum value of the data that are within 1.5 times the interquartile range over the 25th and the 75th percentile respectively.

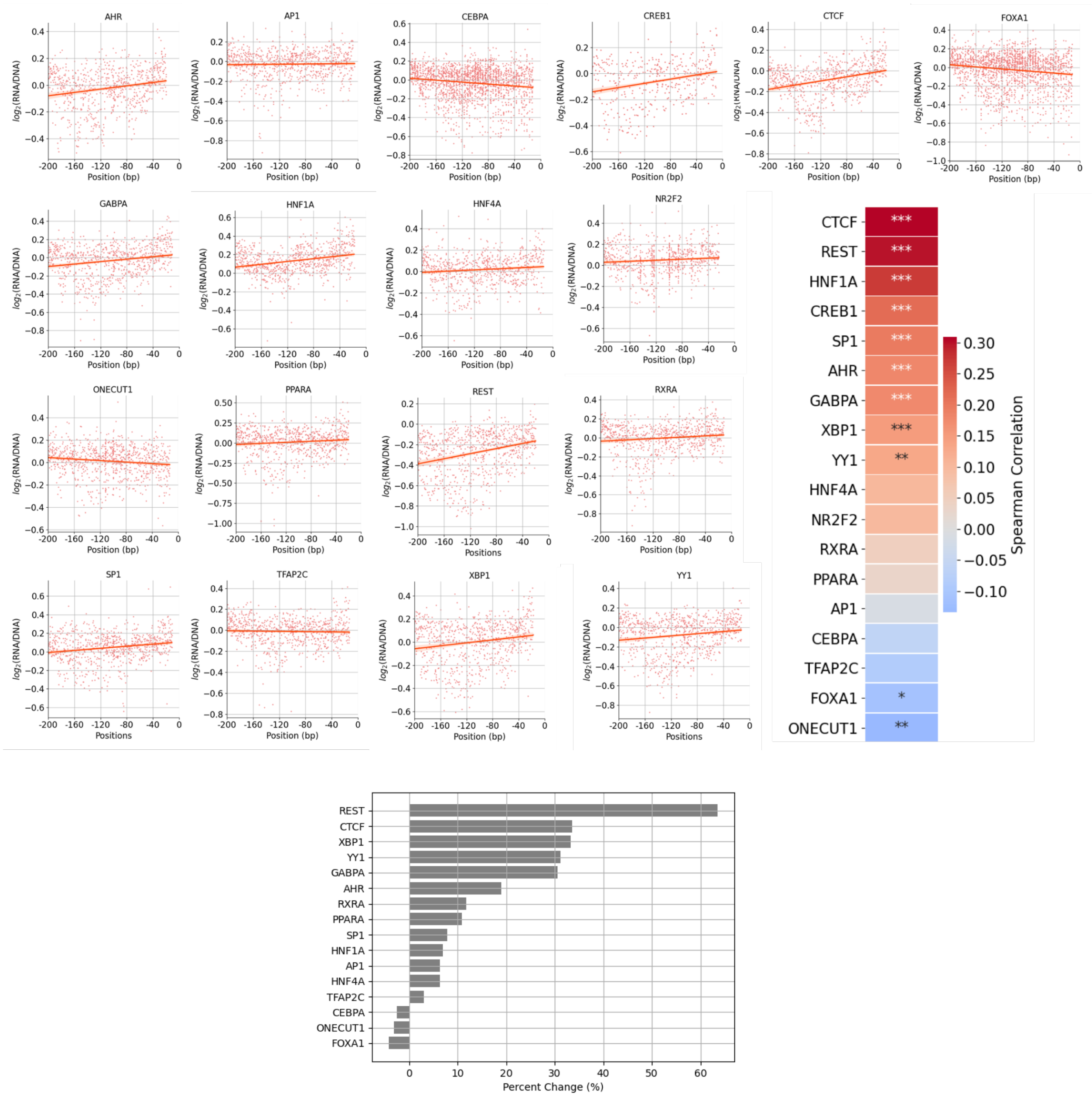

Supplementary Figure 4. Association between position of a single TFBS and expression levels. Significant positive correlations were observed for CTCF, REST, HNF1A, CREB1, SP1, AHR, GABPA, XBP1 and YY1; significant negative correlations were observed for FOXA1 and ONECUT1 (Spearman correlations, Bonferroni-corrected p-values, p-values<0.05). CREB1 is palindromic so there were two occurrences in all instances that were presented instead of a single occurrence in this figure. Percent change in expression for the position of the TFBS in the most proximal (-200bp to -160bp) and most distal bin (-40bp to 0bp) as defined in Figure 2a-b.

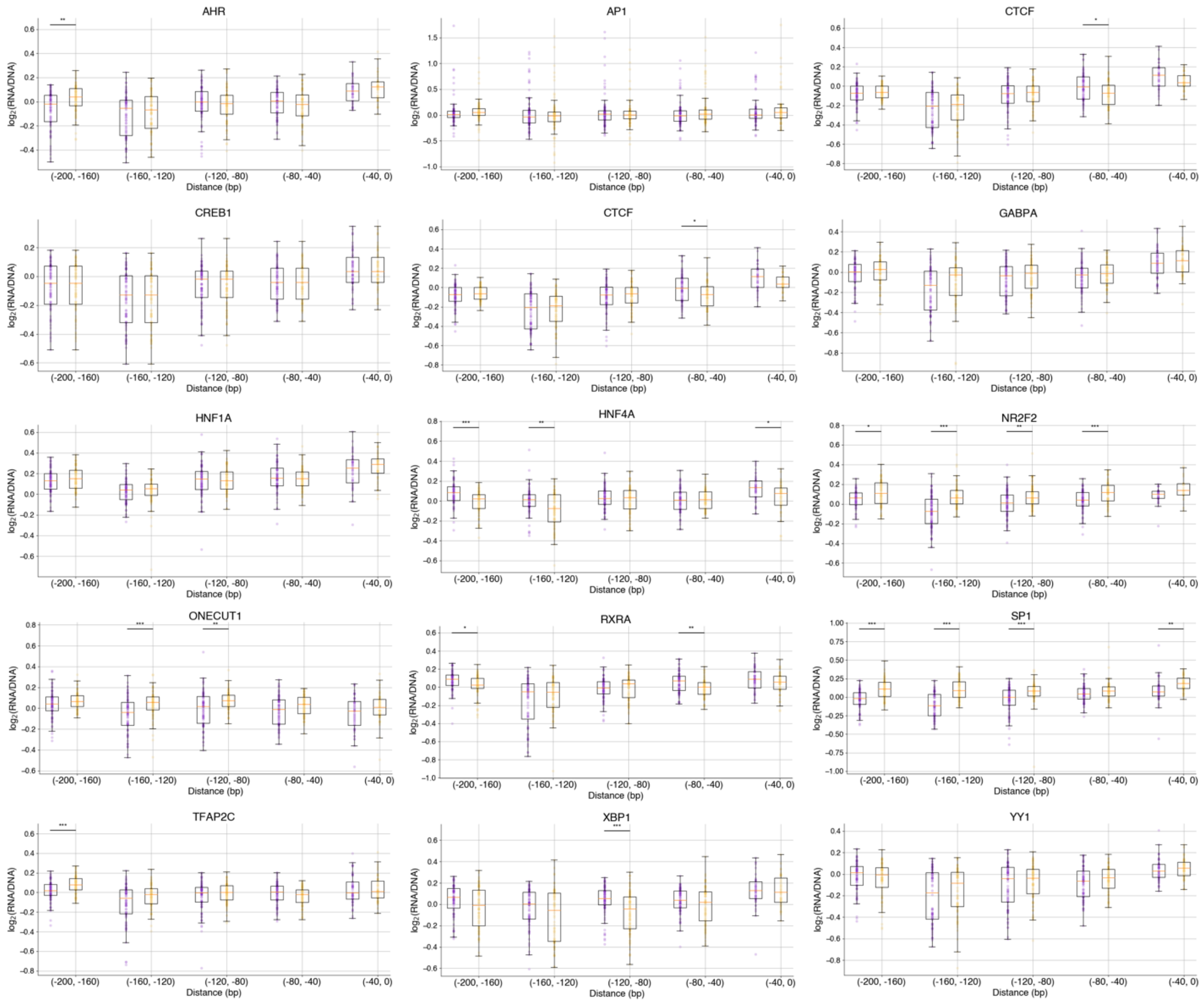

Supplementary Figure 5. Association between the position of TFBSs in the MPRA and expression levels. The template (yellow) and non-template (purple) orientations are shown separately for each TFBS. Boxplot binning the positions of TFBSs for sequences with at least one occurrence of homotypic TFBSs and associated expression levels. Statistical significance was estimated with t-tests with Bonferroni-corrected p-values. Results obtained from n=2 background sequences. In the boxplots, the median is indicated as the center line, the lower and upper limits of the boxplots indicate the first quantile (25th percentile) and the third quantile (75th percentile) respectively, the lower and upper whiskers are the lowest and the maximum value of the data that are within 1.5 times the interquartile range over the 25th and the 75th percentile respectively.

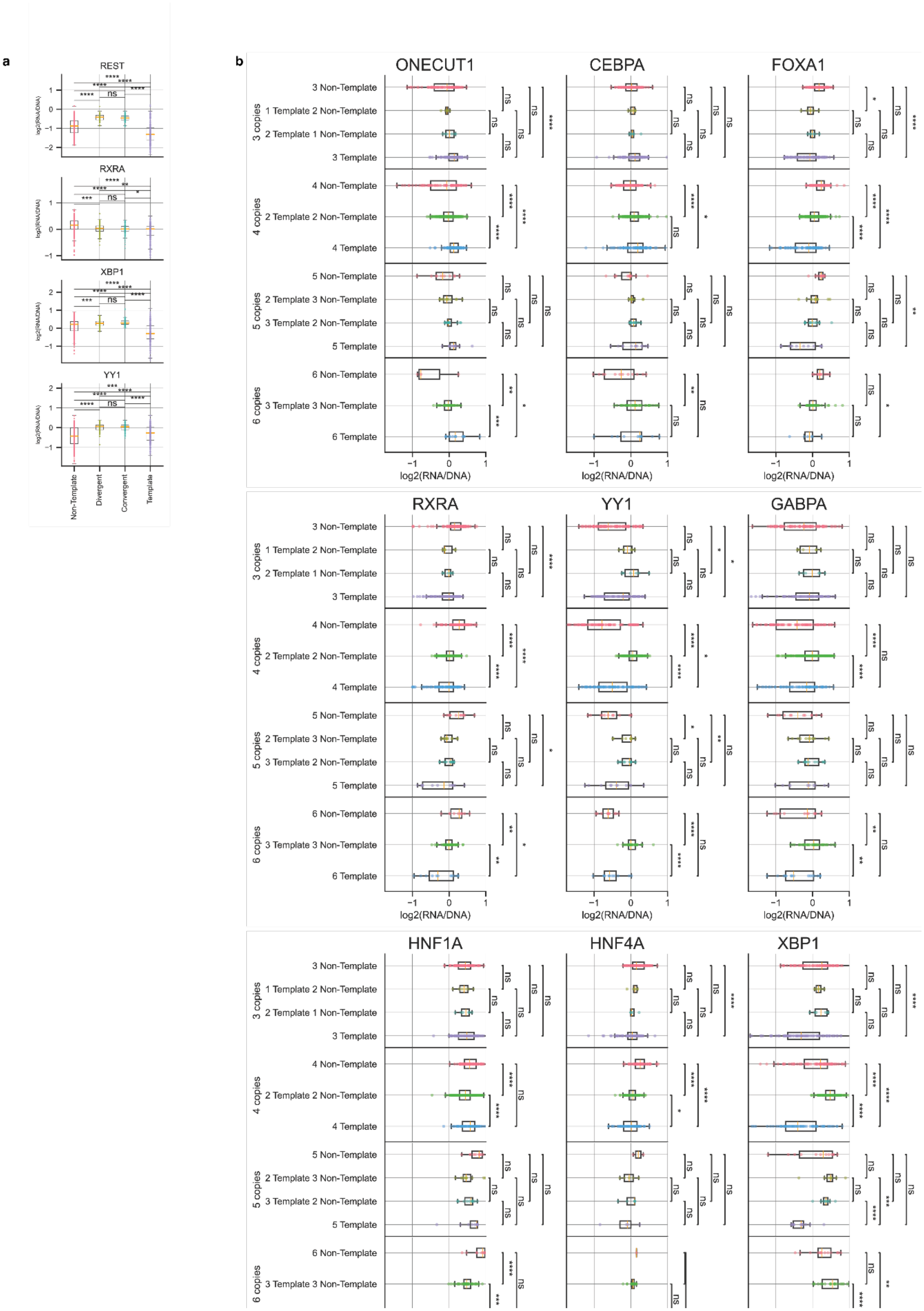
a
REST
RXRA
XBP1
YY1
log2(RNA/DNA)
Non-Template
Divergent
Convergent
Template
b
ONECUT1
CEBPA
FOXA1
RXRA
YY1
GABPA
HNF1A
HNF4A
XBP1
3 copies
4 copies
5 copies
6 copies
3 Non-Template
1 Template 2 Non-Template
2 Template 1 Non-Template
3 Template
4 Non-Template
2 Template 2 Non-Template
4 Template
5 Non-Template
2 Template 3 Non-Template
3 Template 2 Non-Template
5 Template
6 Non-Template
3 Template 3 Non-Template
6 Template
log2(RNA/DNA)

Supplementary Figure 6. Expression differences of homotypic TFBS clusters depending on motif orientation. **a**, Expression levels for homotypic TFBS pairs with both copies in non-template or template orientations or convergent or divergent orientations. Examples include REST, RXRA, XBP1 and YY1displaying statistically significant bias in the expression between any two of the four possible orientations. **b,** Expression difference between orientations for three, four, five and six copies for RXRA, YY1, GABPA, ONECUT1, CEBPA, FOXA1, HNF1A, HNF4A and XBP1. Statistical significance was estimated with t-tests. Adjusted p-values from t-tests displayed as * for p-value<0.05, ** for p-value<0.01 and *** for p-value<0.001. Results obtained from n=2 background sequences. In the boxplots, the median is indicated as the center line, the lower and upper limits of the boxplots indicate the first quantile (25th percentile) and the third quantile (75th percentile) respectively, the lower and upper whiskers are the lowest and the maximum value of the data that are within 1.5 times the interquartile range over the 25th and the 75th percentile respectively.

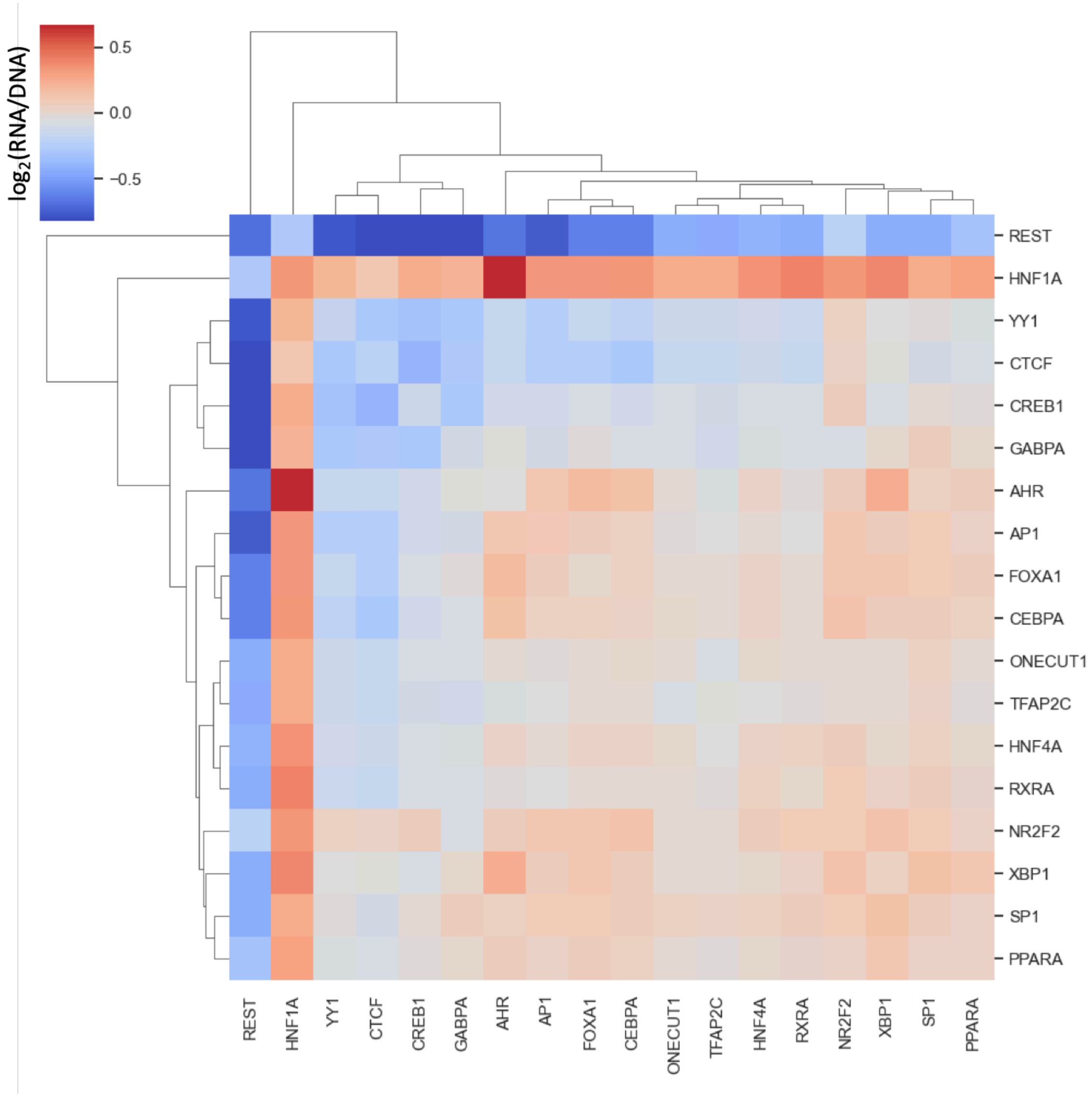

Supplementary Figure 7. Expression levels measured as $\log_2$(RNA/DNA) for TFBS pairs irrespective of order or orientation. The expression levels associated with TFBS pairs is shown, without taking into account individual TFBS orientations (non-template / template) or order (closest / distant) relative to the TSS.

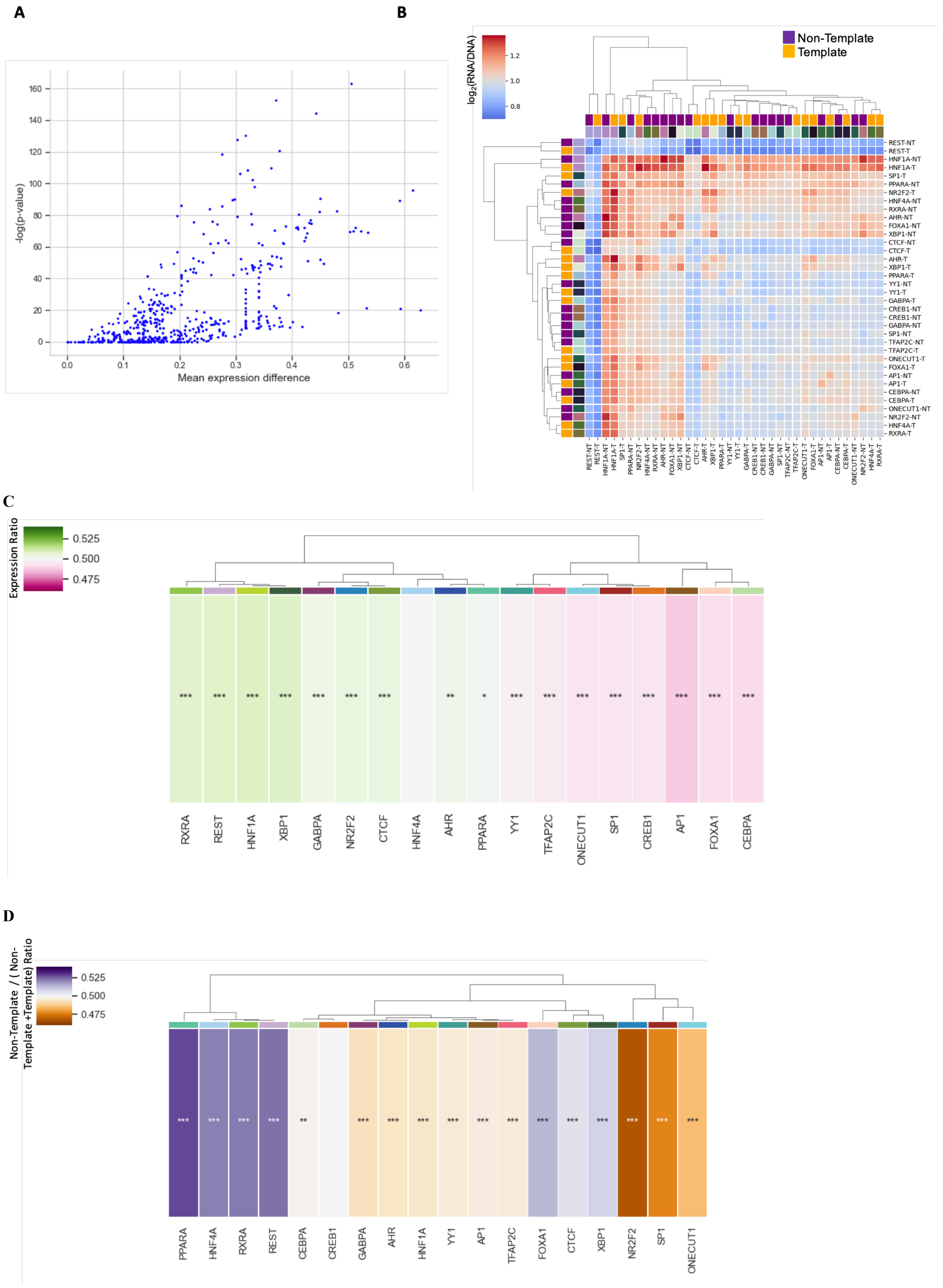
A
B
C
D
-log(p-value)
Mean expression difference
log2(RNA/DNA)
Non-Template
Template
Expression Ratio
Non-Template / (Non-Template +Template) Ratio
0.525
0.500
0.475
RXRA
REST
HNF1A
XBP1
GABPA
NR2F2
CTCF
HNF4A
AHR
PPARA
YY1
TFAP2C
ONECUT1
SP1
CREB1
AP1
FOXA1
CEBPA

Supplementary Figure 8. *Cis*-regulatory effects of orientation and order for heterotypic TFBSs. a, Expression difference depending on the orientations with lowest and highest mean expression with adjusted p-value from t-tests also shown for heterotypic TFBS pairs. **b,** Expression levels depending on orientation across TFBS pairs. **c,** Expression ratio if the TFBS is in the "Distant" over the "Closest" position across heterotypic TFBSs. Expression ratio calculated as mean expression in distant over mean expression in closest and distant positions. **d,** Ratio of expression if the TFBS is in the Non-template over the Non-Template and Template orientation across the heterotypic TFBSs. Adjusted p-values with t-tests displayed as * for p-value<0.05, ** for p-value<0.01 and *** for p-value<0.001.

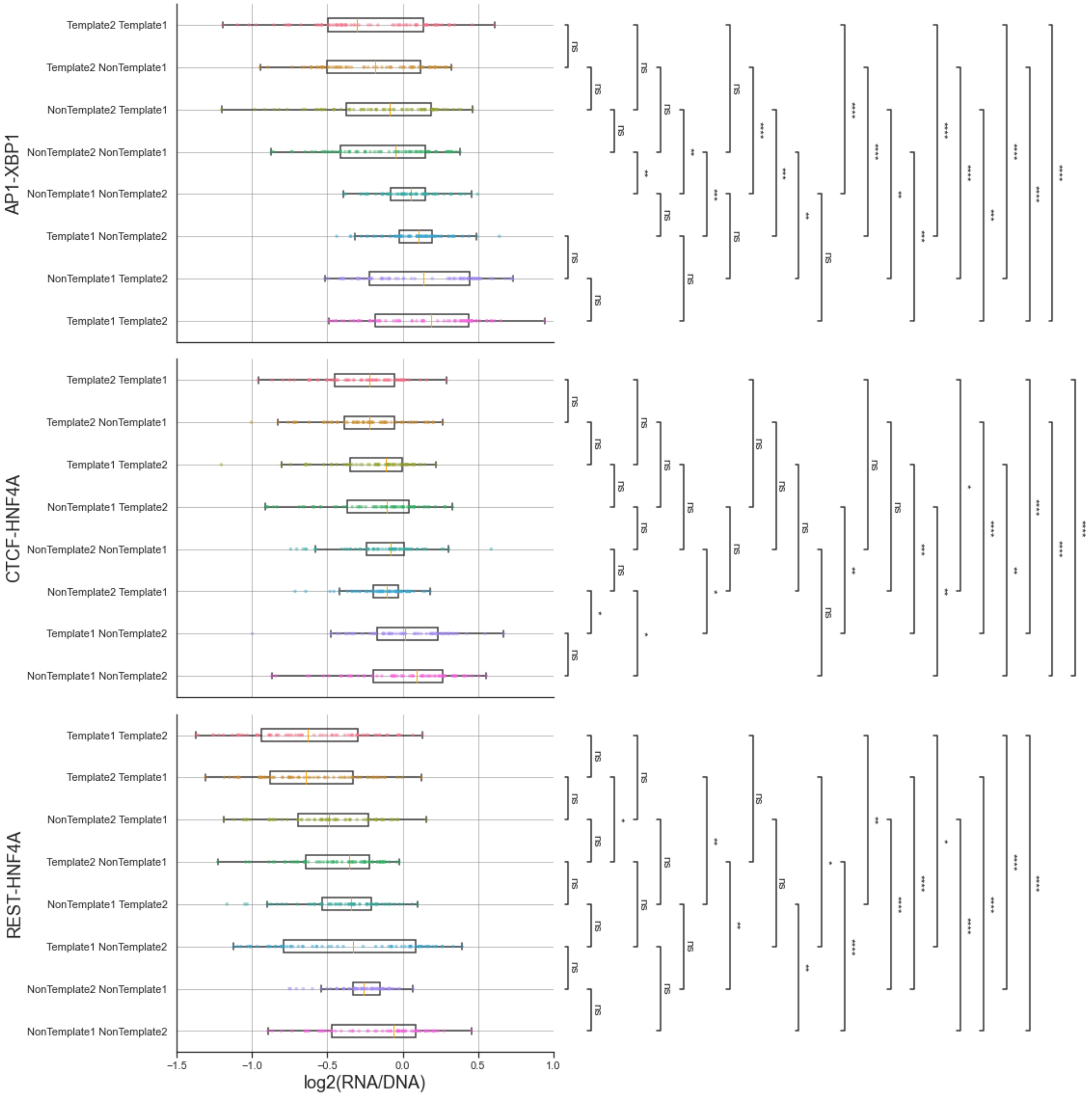

Supplementary Figure 9. The proportion of heterotypic TFBS copies in each orientation influences proportionally expression levels. Template or non-template and one and two refer to the strand and order with which the two TFBSs appear relative to the TSS, with first and second being the TFBSs that appear first and second respectively in the title of the diagram. Results shown for AP1-XBP1, CTCF-HNF4A and REST-HNF4A. Statistical significance was estimated with t-tests and Bonferroni-corrected p-values. Adjusted p-values displayed as * for p-

value<0.05, ** for p-value<0.01 and *** for p-value<0.001. Non-significance is displayed as ns. Results obtained from n=2 background sequences. In the boxplots, the median is indicated as the center line, the lower and upper limits of the boxplots indicate the first quantile (25th percentile) and the third quantile (75th percentile) respectively, the lower and upper whiskers are the lowest and the maximum value of the data that are within 1.5 times the interquartile range over the 25th and the 75th percentile respectively.

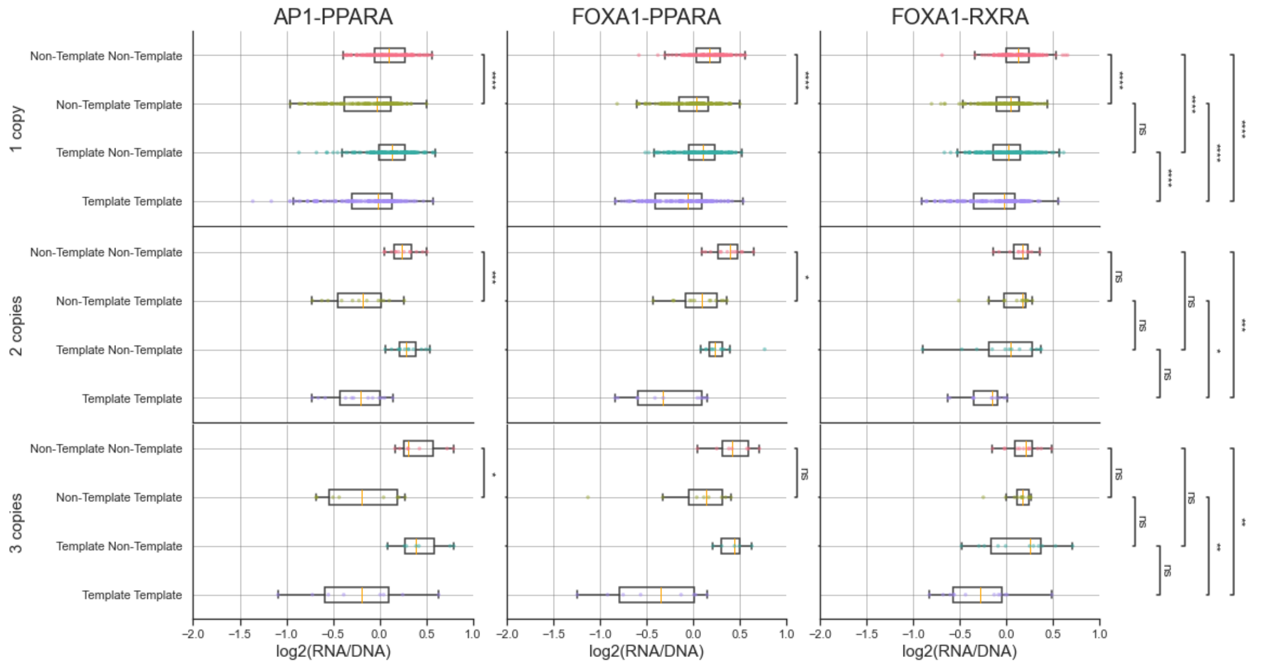

Supplementary Figure 10. Dosage-dependent effects of the proportion of template (T) and non-template (NT) TFBSs in heterotypic pairs. Results shown for one, two or three consecutive copies of each TFBS in the TFBS pair. Three heterotypic TFBS pairs are shown: AP1-PPARA, FOXA1-PPARA, FOXA1-RXRA. Statistical significance was estimated with t-tests and Bonferroni-corrected p-values. Adjusted p-values displayed as * for p-value<0.05, ** for p-value<0.01 and *** for p-value<0.001. Non-significance is displayed as ns. Results obtained from n=2 background sequences. In the boxplots, the median is indicated as the center line, the lower and upper limits of the boxplots indicate the first quantile (25th percentile) and the third quantile (75th percentile) respectively, the lower and upper whiskers are the lowest and the maximum value of the data that are within 1.5 times the interquartile range over the 25th and the 75th percentile respectively.

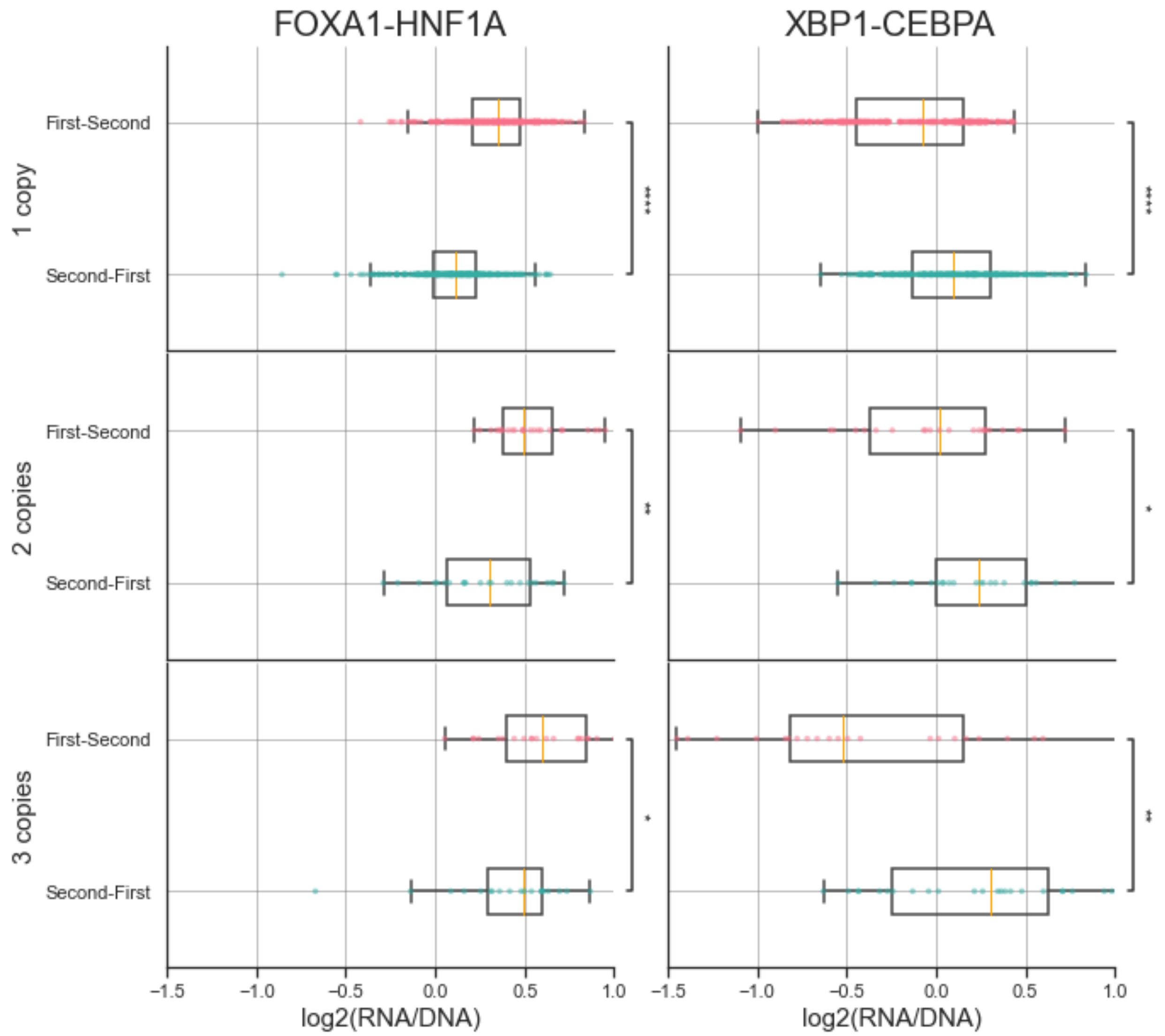

Supplementary Figure 11. The order of heterotypic TFBSs influences expression levels. First and second refer to the order with which the two TFBSs appear relative to the TSS, with first and second being the TFBSs that appear first and second respectively in the title of the diagram. Results shown for one, two or three consecutive copies of the two heterotypic TFBSs. Two pairs are shown for FOXA1-HNF1A and XBP1-CEBPA. Statistical significance was estimated with t-tests and Bonferroni-corrected p-values. Adjusted p-values displayed as * for p-value<0.05, ** for p-value<0.01 and *** for p-value<0.001. Non-significance is displayed as ns. Results obtained from n=2 background sequences. In the boxplots, the median is indicated as the center line, the lower and upper limits of the boxplots indicate the first quantile (25th percentile) and the third quantile (75th percentile) respectively, the lower and upper whiskers are the lowest and the maximum value of the data that are within 1.5 times the interquartile range over the 25th and the 75th percentile respectively.

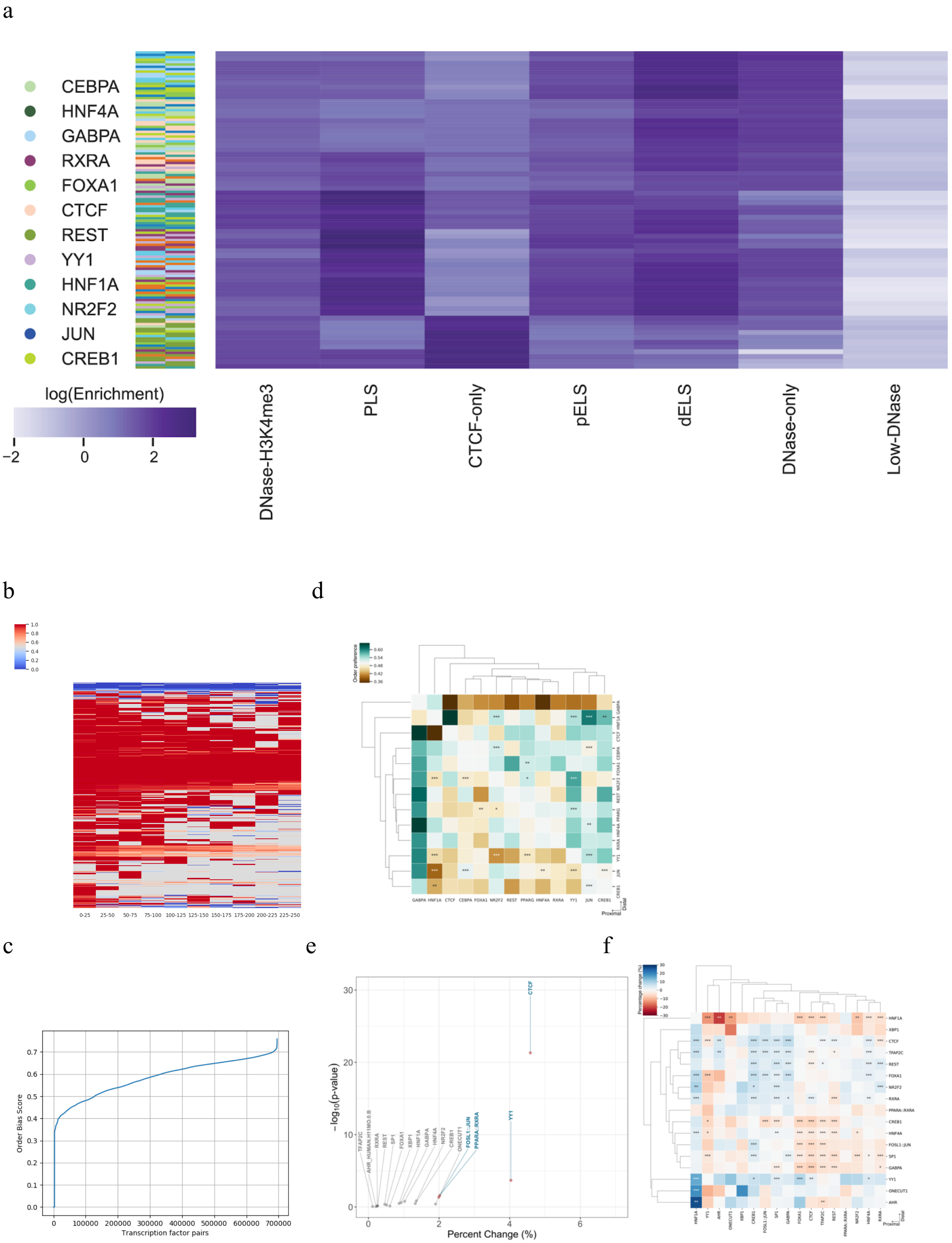

Supplementary Figure 12. TFBSs and TFBS pairs are distributed in the human genome and in promoters with orientation and order biases. a, Enrichment of ChIP-seq bound TFBS pairs (within 100bp from each other) within

putative cis-regulatory elements. **b,** Homotypic occurrences of TFBSs are more frequently found in the same orientation across the human genome. **c,** Order bias for pairs of TFBSs across human promoters. Order bias score was calculated as the proportion of occurrences in one of the two orders (the minimum), **d,** The order preference of ChIP-seq bound TFBS pairs between the promoter-proximal and promoter-distal positions within promoter regions. **e,** Percent change in expression for TFBSs in template and non-template orientations in a HepG2 MPRA experiment. For CTCF, YY1, FOXA1, AP1 (FOSL1::JUN), PPARA:RXRA and HNF4A the results were consistent with the other MPRA performed (Figure1). **f,** The percent change between the two possible orders of heterotypic TFBSs were calculated and shown in the heatmap. In the columns and rows of the heatmap, the proximal and distal transcription factor are displayed respectively. Adjusted p-values displayed as * for p-value<0.05, ** for p-value<0.01 and *** for p-value<0.001.

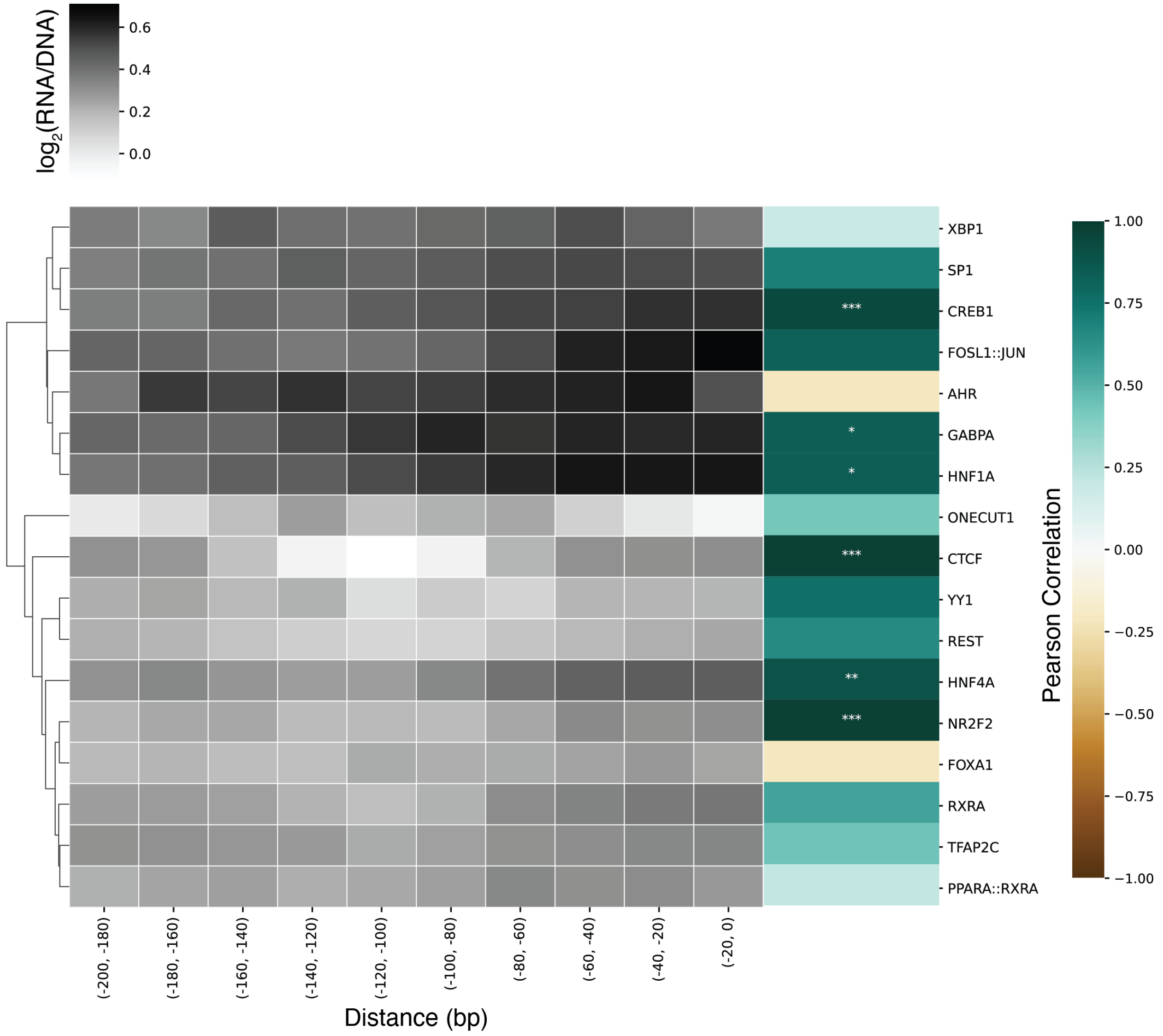

Supplementary Figure 13. Association between the position in the MPRA tile and expression levels for each of the TFBSs. The Pearson correlation is estimated between the position and expression levels and p-value is calculated and is Bonferroni corrected. Adjusted p-values displayed as * for p-value<0.05, ** for p-value<0.01 and *** for p-value<0.001.

a

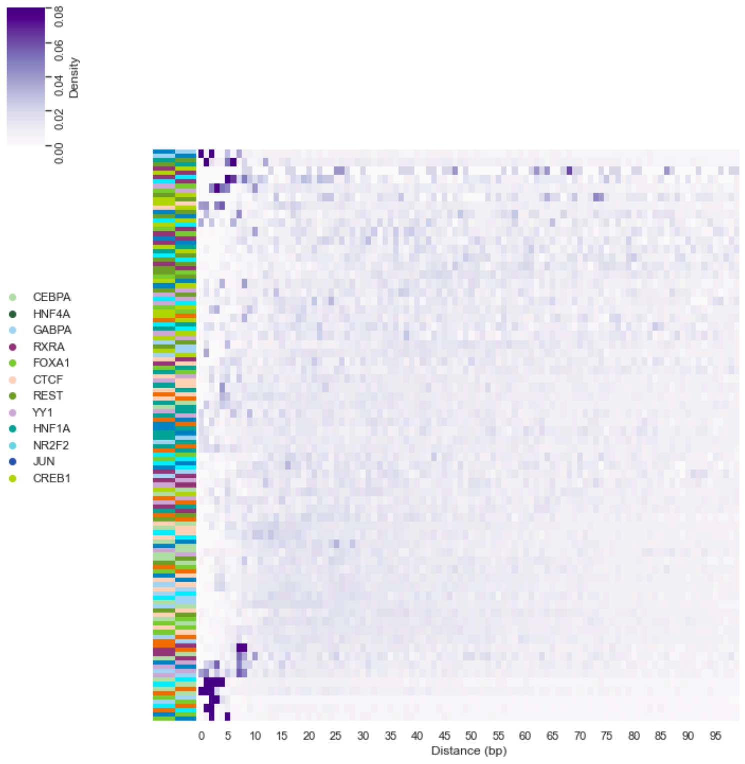

Density
0.00
0.02
0.04
0.06
0.08
CEBPA
HNF4A
GABPA
RXRA
FOXA1
CTCF
REST
YY1
HNF1A
NR2F2
JUN
CREB1
0 5 10 15 20 25 30 35 40 45 50 55 60 65 70 75 80 85 90 95
Distance (bp)

b

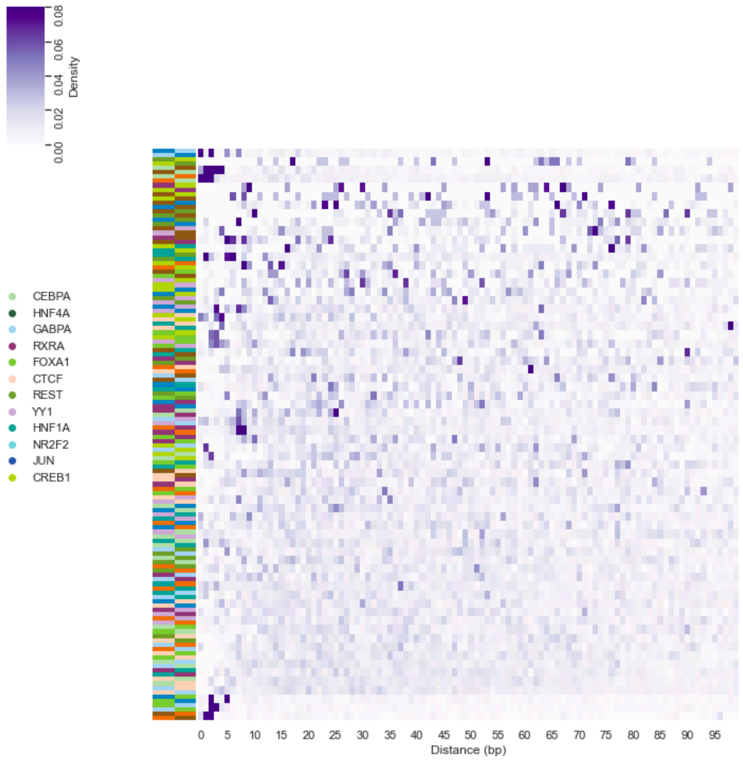

Density
0.00
0.02
0.04
0.06
0.08
CEBPA
HNF4A
GABPA
RXRA
FOXA1
CTCF
REST
YY1
HNF1A
NR2F2
JUN
CREB1
0 5 10 15 20 25 30 35 40 45 50 55 60 65 70 75 80 85 90 95
Distance (bp)

Supplementary Figure 14. Identification of preferred distances between pairs of ChIP-seq bound TFBSs. a, Density of observed distances between pairs of ChIP-seq bound TFBS, within 100bp from each other genome-wide. **b,** Density of observed distances between pairs of ChIP-seq bound TFBS, within 100bp from each other genome-wide.

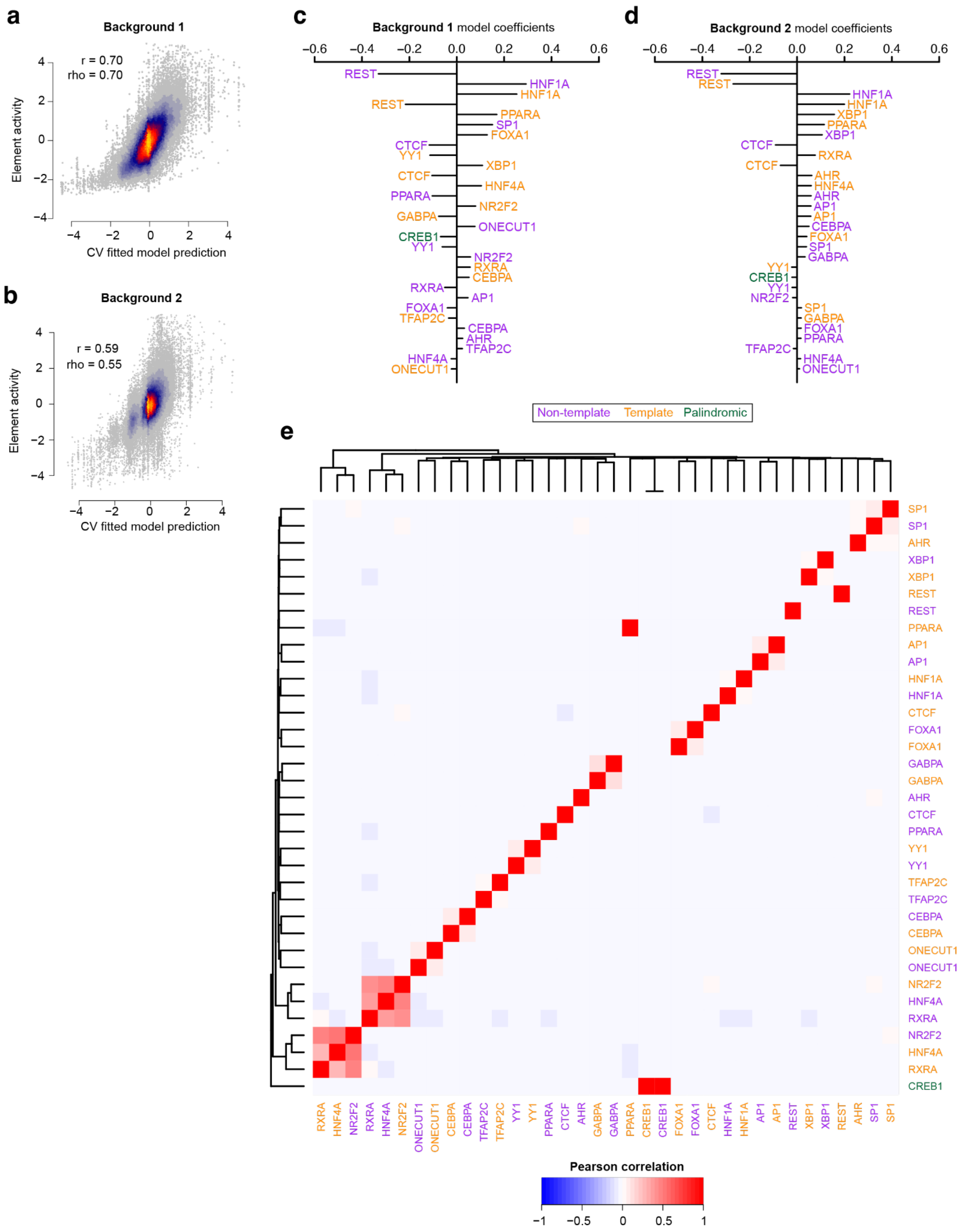

Supplementary Figure 15. A predictive model that accounts for TFBS orientation shows improved performance with both background sequences (constructs) tested. a-b, Plot of the Pearson correlation values for predictive models that are aware of the orientation of TFBSs for background sequence 1 (**a**) and background sequence 2 (**b**). Regions are colored according to the density of data from light blue (low density) to yellow (high density). **c-d,** The

top thirty coefficients relating to orientation-specific TFBSs derived from lasso regression models trained on the full datasets for background sequence 1 (**c**) and background sequence 2 (**d**). **e,** Pearson correlation matrix between the union of all top 30 features from (**c-d**). Features are colored according to the orientation of the TFBS in the following categories: i) non-template, ii) template, iii) palindromic.

Supplementary Table 1. Transcription factors binding sites used in this study. Transcription factors binding sites used were either general or key regulators in the liver and a kmer motif was used throughout the study which is displayed.

Transcription Factor	Type	Sequence Motif	Reference
CTCF	general	CGGCCACCAGGGGGCGCCA	[20]
REST	general	GCGCTGTCCGTGGTGCTGA	[20]
YY1	general	CAAGATGGCGGC	[20]
SP1	general	CCCCGCCCCC	[20]
AP1	general	TGACTCA	[42]
CREB1	general	TGACGTCA	[43]
GABPA	general	CCCGGAAGTG	[20]
RXRA	key regulator in the liver	CAAAGGTCAGG	[20]
FOXA1	key regulator in the liver	TGTTTGCTTTG	[19]
ONECUT1	key regulator in the liver	CAAAATCAATAA	[19]
AHR	key regulator in the liver	GGGGATCGCGTGCCAGCCC	[19]
HNF1A	key regulator in the liver	AGTTAATGATTAACCAA	[19]
NR2F2	key regulator in the liver	CCCCCTGACCTTTGCCCCCTGCC	[19]
PPARA	key regulator in the liver	CCGGGTCATTGGGGTCAGG	[19]
TFAP2C	key regulator in the liver	TGCCCCAAGGCA	[20]
CEBPA	key regulator in the liver	GTTGCACAATA	[20]
XBP1	key regulator in the liver	GTGATGACGTGTCCCAT	[19]
HNF4A	key regulator in the liver	GGGGCAAAGGTCA	[44]

Supplementary Table 2. Oligonucleotide sequences used for the luciferase assays.

Name	Coordinates (hg19)	Sequence
chr2_168	>chr2:211153238–211153405	CTCACTCAGCCTGCATTTCTGCCAGGGCCCGCTCTAGACCTGCAGGAGGACCGGATCAACTTTTTGCCAGCTGGTGTAGATGTTAAAAATTACTGTCACTCTTCCGCCTGCTACTTTATTTTGCACCTGCTGTTACTTGAGTTACAGGCATTTCACACATGGTAATTTAATAAGGTTAGTTCCCATGACAATGTACTAGATATTATCCCATTGAGGGTTAAAGTGGTTCATTGCGTGAACCGAGGCACTAGAGGGTATATAATGGAAGCTCGACTTCCAGCTTGGCAATCCGGTACtgt
chr2_200	>chr2:211153222-211153422	CTCACTCAGCCTGCATTTCTGCCAGGGCCCGCTCTAGACCTGCAGGAGGACCGGATCAACTATTAGGCACCTGTCATTTTTGCCAGCTGGTGTAGATGTTAAAAATTACTGTCACTCTTCCGCCTGCTACTTTATTTTGCACCTGCTGTTACTTGAGTTACAGGCATTTCACACATGGTAATTTAATAAGGTTAGTTCCCATGACAATGTACTAGATATTATCCCATTGAGGGTTAAAGTGGTTTATAAGGTCTTGAGGGCATTGCGTGAACCGAGGCACTAGAGGGTATATAATGGAA
chr9_168	>chr9:83712599-83712766	CTCACTCAGCCTGCATTTCTGCCAGGGCCCGCTCTAGACCTGCAGGAGGACCGGATCAACTTGTTCAGAAGGGCCAGAAATGCCAAGGACTCAGGGGAGGAGAATTAAGTCAGAGAGTTTCATTACTGAGTGTTGTTTGACTTTGTTGTCACGGATTCATTTAACCATCTCTCTACCATGGTAAAAATGTGTATCCTATGTCCAGTATGAAATAAAAACTGCCTCCTTCCATTGCGTGAACCGAGGCACTAGAGGGTATATAATGGAAGCTCGACTTCCAGCTTGGCAATCCGGTACtgt
chr9_200	>chr9:83712583-83712783	CTCACTCAGCCTGCATTTCTGCCAGGGCCCGCTCTAGACCTGCAGGAGGACCGGATCAACTGCAAGTCTGCCATCGTGTTCAGAAGGGCCAGAAATGCCAAGGACTCAGGGGAGGAGAATTAAGTCAGAGAGTTTCATTACTGAGTGTTGTTTGACTTTGTTGTCACGGATTCATTTAACCATCTCTCTACCATGGTAAAAATGTGTATCCTATGTCCAGTATGAAATAAAAACTGCCTCCTTCCAAATTAGAGGTGGCTgCATTGCGTGAACCGAGGCACTAGAGGGTATATAATGGAA
Negative	>chr2:238336485-238336655	CTCACTCAGCCTGCATTTCTGCCAGGGCCCGCTCTAGACCTGCAGGAGGACCGGATCAACTGTTCACTCCCACTTCTCAGACACGGACATTACGCAGCTCCAAGGGAGTTAGGACTCAGCAGCTGAAAACCACCAATTCATGCCTCATCAGTGTCAATGTCAGTCCCCACACTTGTACTTCCAGATGTCTATGAGGTTGTGCCAAGCAGCCCCTCTCACATGCCTTACCACTCATTGCGTGAACCGAGGCACTAGAGGGTATATAATGGAAGCTCGACTTCCAGCTTGGCAATCCGGTAC
