# Supplementary material for: Transcription factor binding site orientation and order are major drivers of gene regulatory activity
Source: Nat Commun. 2023 Apr 22;14:2333. doi: 10.1038/s41467-023-37960-5 (PMC10122648; doi:10.1038/s41467-023-37960-5)
Supplement: Supplementary file 2 — Additional Supplementary Files [file 41467_2023_37960_MOESM2_ESM.pdf]

Description of Additional Supplementary Files

File Name: Supplementary Data 1
Description: The orientation of TFBSs in heterotypic TFBS pairs impacts expression levels.
