# Supplementary material for: Transcription factor binding site orientation and order are major drivers of gene regulatory activity
Source: Nat Commun. 2023 Apr 22;14:2333. doi: 10.1038/s41467-023-37960-5 (PMC10122648; doi:10.1038/s41467-023-37960-5)
Supplement: Supplementary file 4 — Reporting Summary [file 41467_2023_37960_MOESM4_ESM.pdf]

nature portfolio

Corresponding author(s): Ilias Georakopoulos-Soares, Nadav Ahituv

Last updated by author(s): Mar 16, 2023

Reporting Summary

Nature Portfolio wishes to improve the reproducibility of the work that we publish. This form provides structure for consistency and transparency in reporting. For further information on Nature Portfolio policies, see our Editorial Policies and the Editorial Policy Checklist.

Statistics

For all statistical analyses, confirm that the following items are present in the figure legend, table legend, main text, or Methods section.

n/a	Confirmed	
☐	☒	The exact sample size (*n*) for each experimental group/condition, given as a discrete number and unit of measurement
☐	☒	A statement on whether measurements were taken from distinct samples or whether the same sample was measured repeatedly
☐	☒	The statistical test(s) used AND whether they are one- or two-sided<br>*Only common tests should be described solely by name; describe more complex techniques in the Methods section.*
☐	☒	A description of all covariates tested
☐	☒	A description of any assumptions or corrections, such as tests of normality and adjustment for multiple comparisons
☐	☒	A full description of the statistical parameters including central tendency (e.g. means) or other basic estimates (e.g. regression coefficient) AND variation (e.g. standard deviation) or associated estimates of uncertainty (e.g. confidence intervals)
☐	☒	For null hypothesis testing, the test statistic (e.g. *F*, *t*, *r*) with confidence intervals, effect sizes, degrees of freedom and *P* value noted<br>*Give P values as exact values whenever suitable.*
☐	☒	For Bayesian analysis, information on the choice of priors and Markov chain Monte Carlo settings
☐	☒	For hierarchical and complex designs, identification of the appropriate level for tests and full reporting of outcomes
☐	☒	Estimates of effect sizes (e.g. Cohen's *d*, Pearson's *r*), indicating how they were calculated

Our web collection on statistics for biologists contains articles on many of the points above.

Software and code

Policy information about availability of computer code

Data collection	No software was used.
Data analysis	MPRA was conducted with three independent replicates and analyzed using MPRAflow.<br>Position frequency matrices (PFMs) of transcription factors were inserted into FIMO for finding TFBSs.<br>Identification of TFBSs at promoters was performed with BEDTools intersect function.<br>We aligned all consensus sequences back to all designed sequences (inserts) using BWA MEM (version 0.7.17-r1188).

For manuscripts utilizing custom algorithms or software that are central to the research but not yet described in published literature, software must be made available to editors and reviewers. We strongly encourage code deposition in a community repository (e.g. GitHub). See the Nature Portfolio guidelines for submitting code & software for further information.

Data

Policy information about availability of data

All manuscripts must include a data availability statement. This statement should provide the following information, where applicable:

- Accession codes, unique identifiers, or web links for publicly available datasets
- A description of any restrictions on data availability
- For clinical datasets or third party data, please ensure that the statement adheres to our policy

Position frequency matrices (PFMs) of transcription factors were derived from JASPAR (release 2022) for the non-redundant CORE vertebrate collection (http://

jaspar.genereg.net/download/CORE/JASPAR2022_CORE_vertebrates_non-redundant_pfms_meme.zip). A lentiMPRA dataset that has been generated by our group as part of the ENCODE consortium (Accession: "ENCSR359FTN [https://www.encodeproject.org/references/ENCSR359FTN/]") of 164,307 sequences tested was analyzed.
RNA-seq data from HepG2 were derived from the Roadmap Epigenomics Consortium using the processed FPKM expression matrix. The lentiMPRA data generated in this study have been deposited in the GEO database under accession code PRJNA854975. Source data are provided with this paper.

For the eighteen transcription factors in the MPRA, for identifying the optimal distance between them, the following sequences were used: for AP1 the motif TGACTCA, for CREB1 the motif TGACGTCA and the PWMs with the following JASPAR IDs: "MA0102.3 [https://jaspar.genereg.net/matrix/MA0102.3/]" (CEBPA), "MA0139.1 [https://jaspar.genereg.net/matrix/MA0139.1/]" (CTCF), "MA0148.3 [https://jaspar.genereg.net/matrix/MA0148.3/]" (FOXA1), "MA0062.2 [https://jaspar.genereg.net/matrix/MA0062.2/]" (GABPA), "MA0046.2 [https://jaspar.genereg.net/matrix/MA0046.2/]" (HNF1A), "MA0114.3 [https://jaspar.genereg.net/matrix/MA0114.3/]" (HNF4A), "MA1111.1 [https://jaspar.genereg.net/matrix/MA1111.1/]" (NR2F2), "MA0679.1 [https://jaspar.genereg.net/matrix/MA0679.1/]" (ONECUT1), "MA1148.1 [https://jaspar.genereg.net/matrix/MA1148.1/]" (PPARA_RXRA), "MA0138.2 [https://jaspar.genereg.net/matrix/MA0138.2/]" (REST), "MA0512.2 [https://jaspar.genereg.net/matrix/MA0512.2/]" (RXRA), "MA0079.3 [https://jaspar.genereg.net/matrix/MA0079.3/]" (SP1), "MA0524.2 [https://jaspar.genereg.net/matrix/MA0524.2/]" (TFAP2C), "MA0844.1 [https://jaspar.genereg.net/matrix/MA0844.1/]" (XBP1), "MA0095.2 [https://jaspar.genereg.net/matrix/MA0095.2/]" (YY1) and "MA0148.3 [https://jaspar.genereg.net/matrix/MA0148.3/]" (FOXA1). For AHR, the HOCOMOCO PWM was used: "AHR_HUMAN.H11MO.0 [https://hocomoco11.autosome.org/motif/AHR_HUMAN.H11MO.0.B]".
ChIP-seq bound TFBS were derived from UniBind25 for CTCF ("ENCSR000AMA [https://www.encodeproject.org/experiments/ENCSR000AMA/]"), CREB1 ("ENCSR000BVL [https://www.encodeproject.org/experiments/ENCSR000BVL/]"), FOXA1 ("ENCSR000BLE [https://www.encodeproject.org/experiments/ENCSR000BLE/]"), GABPA ("ENCSR000BJK [https://www.encodeproject.org/experiments/ENCSR000BJK]"), HNF1A "ENCSR800QIT ("[https://www.encodeproject.org/experiments/ENCSR800QIT]"), HNF4A ("ENCSR000BLF [https://www.encodeproject.org/experiments/ENCSR000BLF/]"), JUN ("ENCSR000EEK [https://www.encodeproject.org/experiments/ENCSR000EEK/]"), NR2F2 ("ENCSR000BVM [https://www.encodeproject.org/experiments/ENCSR000BVM/]"), REST ("ENCSR000BOT [https://www.encodeproject.org/experiments/ENCSR000BOT/]"), RXRA ("ENCSR000BHU [https://www.encodeproject.org/experiments/ENCSR000BHU/]") and YY1 ("ENCSR000BNT [https://www.encodeproject.org/experiments/ENCSR000BNT/]").

Human research participants

Policy information about studies involving human research participants and Sex and Gender in Research.

Reporting on sex and gender	NA
Population characteristics	NA
Recruitment	NA
Ethics oversight	NA

Note that full information on the approval of the study protocol must also be provided in the manuscript.

Field-specific reporting

Please select the one below that is the best fit for your research. If you are not sure, read the appropriate sections before making your selection.

☒ Life sciences ☐ Behavioural & social sciences ☐ Ecological, evolutionary & environmental sciences

For a reference copy of the document with all sections, see nature.com/documents/nr-reporting-summary-flat.pdf

Life sciences study design

All studies must disclose on these points even when the disclosure is negative.

Sample size	A lenti-MPRA of209,440 sequences was generated. Sample size was determined based on having two templates, and generating all combinations and permutations of 18 general and liver-specific TFs.
Data exclusions	No data were excluded.
Replication	Three different replicates were used and all of them were successful.
Randomization	Sequences were grouped based on orientation, order and position of TFBSs in the MPRA constructs. Orientation groups were template and non-template orientations for TFBSs. Order groups were based on proximal or distal position relative to the TSS. Orientation accounts for nucleotide composition so it served to compare against the two orientations. Distance effects were examined with correlation tests from distance from TSS.
Blinding	Blinding was not relevant, since we were examining the cis-regulatory code.

Reporting for specific materials, systems and methods

We require information from authors about some types of materials, experimental systems and methods used in many studies. Here, indicate whether each material, system or method listed is relevant to your study. If you are not sure if a list item applies to your research, read the appropriate section before selecting a response.

Materials & experimental systems

n/a	Involved in the study
☒	☐ Antibodies
☐	☒ Eukaryotic cell lines
☒	☐ Palaeontology and archaeology
☒	☐ Animals and other organisms
☒	☐ Clinical data
☒	☐ Dual use research of concern

Methods

n/a	Involved in the study
☒	☐ ChIP-seq
☒	☐ Flow cytometry
☒	☐ MRI-based neuroimaging

Eukaryotic cell lines

Policy information about cell lines and Sex and Gender in Research

Cell line source(s)	HepG2 cells were purchased from Cell and Genome Engineering Core at UCSF (STR profile validated) and were tested negative for mycoplasma using Universal Mycoplasma Detection Kit (30-1012K, American Type Culture Collection).
Authentication	The cell lines used were not further authenticated besides ordering them from Cell and Genome Engineering Core at UCSF (STR profile validated).
Mycoplasma contamination	HepG2 cells were purchased from Cell and Genome Engineering Core at UCSF (STR profile validated) and were tested negative for mycoplasma using Universal Mycoplasma Detection Kit (30-1012K, American Type Culture Collection).
Commonly misidentified lines (See ICLAC register)	HepG2 was used, which is commonly used in ENCODE, useful for researchers since many other experiments have been performed and can be used togeter.
